# Irisin‐mediated muscle‐renal crosstalk as a protective mechanism against contrast‐induced acute kidney injury via cGAS‐STING signalling inhibition

**DOI:** 10.1002/ctm2.70235

**Published:** 2025-02-26

**Authors:** Long Peng, Suhua Li, Qiang Huang, Yuxiang Sun, Juan Sun, Ting Luo, Yanlin Wang, Zhaoyong Hu, Weiyan Lai, Hui Peng

**Affiliations:** ^1^ Division of Cardiovascular Medicine, Department of Medicine The Third Affiliated Hospital, Sun Yat‐Sen University Guangzhou China; ^2^ Division of Nephrology, Department of Medicine The Third Affiliated Hospital, Sun Yat‐Sen University Guangzhou China; ^3^ Division of Nephrology, Department of Medicine University of Connecticut School of Medicine Farmington Connecticut USA; ^4^ Division of Nephrology, Department of Medicine Baylor College of Medicine Houston Texas USA

**Keywords:** cGAS‐STING, contrast‐induced acute kidney injury, inflammation, irisin, mPGC‐1α

## Abstract

**Background:**

Contrast‐induced acute kidney injury (CI‐AKI) continues to pose a pressing clinical challenge during invasive cardiovascular procedures due to the limited availability of preventative strategies. We aimed to demonstrate that irisin, a myokine induced by exercise, protects against CI‐AKI by inhibiting the cGAS‐STING inflammatory pathway.

**Methods and results:**

We explored the relationship between serum irisin levels and CI‐AKI incidence in patients administered the contrast media iohexol. Notably, lower serum irisin levels were strongly associated with an increased incidence of CI‐AKI following contrast media administration. To establish a causal link between serum irisin levels and CI‐AKI, we utilised a mouse model that simulates exercise by overexpressing muscle‐specific PGC‐1α. This approach showed a significant reduction in tubular injury and mitochondrial dysfunction induced by iohexol via cGAS/STING suppression, thereby diminishing inflammation. Mechanistically, irisin was found to inhibit the activation of cGAS/STING, preventing double stranded DNA (dsDNA) leakage and reducing inflammation in tubular epithelial cells (TECs). Pharmacological inhibition of STING further corroborated these observations. Moreover, we identified integrin complex αV/β5 as the irisin receptor on TECs, which is essential for irisin‐mediated suppression of cGAS‐STING signalling and resolution of inflammation.

**Conclusions:**

Our data position irisin as a crucial factor in muscle‒kidney crosstalk, inhibiting cGAS‐STING signalling and preventing dsDNA leakage via integrin αV/β5 in TECs, thus mitigating tubular injury and inflammation. These data underscore the potential of irisin as both a predictive biomarker for CI‐AKI and a promising candidate for preventative strategies against CI‐AKI.

**Highlights:**

- Irisin mediated muscle‐kidney crosstalk mitigated tubular injury and inflammation.
- Irisin inhibited the cGAS‐STING signalling activation via integrin αV/β5 in tubular epithelial cells.
- Irisin was a predictive biomarker and a promising candidate for CI‐AKI.

## Graphical abstract

Irisin is identified as a crucial factor in muscle‒kidney crosstalk, inhibiting cGAS‐STING signaling and preventing dsDNA leakage via integrin αV/β5 in tubular epithelial cells (TECs), thus mitigating tubular injury and inflammation.

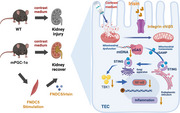

## 1 INTRODUCTION

Contrast‐induced acute kidney injury (CI‐AKI) is increasingly recognised as the leading cause of hospital‐acquired kidney injury.^1^ This condition arises following the administration of contrast agents, commonly used in diagnostic imaging procedures during coronary interventional surgery, and is characterised by a sudden deterioration in renal function. CI‐AKI is associated with severe complications, including acute renal failure, exacerbation of chronic kidney disease (CKD) and cardiovascular events.^2^, ^3^ The pathogenesis of CI‐AKI is multifactorial, involving renal haemodynamic changes, direct tubular epithelial cell (TEC) toxicity and induction of oxidative stress and inflammatory responses. Despite its prevalence and impact, CI‐AKI presents a significant clinical challenge due to the limitation of effective prevention and treatment strategies.^4^, ^5^

Inflammation serves as a crucial pathological hallmark of CI‐AKI, acting as a central initiator of secondary complications.^6^, ^7^ The cyclic GMP‐AMP synthase‐stimulator of the Interferon Genes (cGAS‐STING) pathway has emerged as an important mediator in the inflammatory response.^8^ Activation of this pathway occurs when cytosolic DNA, including viral, bacterial or mitochondrial DNA (mtDNA), binds to cGAS, forming cyclic guanylic acid‐adenylic acid (cGAMP), which activates STING. This activation triggers TANK‐binding kinase 1 (TBK1), leading to the upregulation of interferon expression, and the activation of nuclear factor‐kappa B (NF‐κB) pathway, followed by the release of pro‐inflammatory mediators such as tumour necrosis factor‐alpha (TNF‐α) and interleukin‐6 (IL‐6). An investigation by Maekawa et al. demonstrated that cisplatin‐induced mitochondrial damage in TECs increases mtDNA leakage, activating cGAS‐STING pathway and contributing to renal inflammation.^9^ While studies suggest exercise may have protective effects in kidney injury scenarios such as CKD^10^ its specific influence on the inflammatory processes during AKI is unclear. Particularly, whether exercise‐induced metabolism changes modulating a specific inflammatory pathway, such as cGAS‐STING, has not been explored. Our study aims to bridge this knowledge gap, investigating the unique role of exercise‐induced myokines in CI‐AKI and their potential to modulate critical inflammatory pathways.

Skeletal muscle, through exercise, orchestrates inter‐organ communication by synthesising and releasing myokines such as irisin, IL‐15 and fibroblast growth factor 2.^11^, ^12^, ^13^, ^14^ Irisin is a myokine that is produced from the cleavage of fibronectin type III domain‐containing protein 5 (FNDC5), which is upregulated during physical activity.^15^, ^16^ Irisin plays a pivotal role in various pathophysiological processes, including adipocyte browning, thermogenesis, energy metabolism, insulin resistance, and inflammatory and oxidative stress responses.^14^, ^17^, ^18^, ^19^ Notably, irisin has shown potential in ameliorating memory deficits in Alzheimer's disease models and enhancing endothelial barrier function.^20^, ^21^ Our previous work has indicated that muscle‐specific PGC‐1α (mPGC‐1α) overexpression in mice can modulate energy metabolism reprogramming in injured TECs, potentially delaying CKD progression through irisin secretion.^11^ However, the specific regulation and underlying mechanisms of irisin in muscle‒kidney crosstalk during CI‐AKI remain largely undefined.

This study sought to investigate whether circulating irisin levels could serve as potential predictors of CI‐AKI in patients undergoing cardiac procedures. We also hypothesised that exercise‐induced myokines, particularly irisin, could alleviate CI‐AKI. To investigate this possibility, we used the mPGC‐1α mouse model as a proxy for the physiological effects of exercise on irisin levels. We assessed the activation state of cGAS/STING signalling and inflammatory markers in TECs under CI‐AKI conditions in these mice, comparing them to control groups. Additionally, we conducted in vitro experiments to directly examine the impact of irisin on TECs, particularly its potential role in modulating the cGAS‐STING pathway. These approaches allowed us to explore the cellular mechanisms by which irisin might mitigate CI‐AKI, focusing on the modulation of key signalling pathways involved in tubular inflammation. Through these investigations, our study seeks to bridge the gap in understanding the protective effects of exercise‐induced myokines, such as irisin, in the context of CI‐AKI.

## 2 METHODS

### 2.1 Human study design and sample collection

In cohort study, total 1575 consecutive patients aged 18 years or older admitted to Cardiovascular Department in the Third Affiliated Hospital and Lingnan Hospital of Sun Yat‐Sen University, and scheduled for invasive coronary angiography (CAG) from August 2022 to July 2023. Follow‐up for clinical outcomes was performed 7 days after CAG or percutaneous coronary intervention (PCI) procedure. The exclusion criteria for this study included pregnancy, end‐stage renal failure, a history of allergy to contrast agent, and the use of contrast agent or nephrotoxic drugs within 7 days before enrollment. The research protocol received approval from the Ethics Committee of the Third Affiliated Hospital of Sun Yat‐Sen University (2022‐02‐194‐01), and written informed consent was obtained from all participants. Thus, 290 patients were enrolled in this study (Figure S1). The endpoint was CI‐AKI, defined as a SCr value of 1.5 times the baseline value or ≥.3 mg/dL increase. Blood samples were collected from 73 patients undergoing PCI or CAG procedures, using pyrogen‐free tubes without EDTA. The serum was then separated for irisin quantification.

### 2.2 Animal experiments and therapeutic experiments

mPGC‐1α transgenic C57BL/6 mice were obtained from Jackson Laboratories. The genotype of mPGC‐1α offspring was identified through PCR amplification of DNA derived from tail tissues at 2–3 weeks of age. All experiments were conducted with age‐matched littermates at 7–8 weeks. Direct toxic injury from contrast media to TECs is unlikely in healthy kidneys. Rats and mice require an additional renal insult to develop clinically manifest CI‐AKI. The prostaglandin and nitric oxide synthase (NOS) inhibition models have been reported to closely resemble human CI‐AKI pathology, with injury predominantly localised to the renal medulla.^22^ As previously detailed, the CI‐AKI mouse model was conducted by intraperitoneal administration of iohexol (GE Healthcare). Both wild‐type (WT) and mPGC‐1α mice were deprived of free access to oral water for 72 h before receiving intraperitoneal injections of 10 mg/kg of NG‐nitro‐L‐arginine methyl ester (L‐NAME, an inhibitor of NOS, Sigma), and 10 mg/kg of indomethacin (an inhibitor of prostaglandin synthesis, Sigma). Thirty minutes post‐L‐NAME and indomethacin administration, iohexol was administered intraperitoneally (5 g iodine/kg). Control littermates received saline solution injections. After 24 h, the serum and kidney tissues of mice were collected after terminal anaesthesia by .5% pentobarbital sodium. For H.151 treatment, CI‐AKI mice were pre‐treated with H.151 (750 nmol per mouse, Selleck) daily for 4 days before iohexol treatment via intraperitoneal injection. For irisin therapy, mice were pre‐treated with recombinant irisin (200 µg/kg, R&D Systems) 1 day before iohexol treatment by intraperitoneal injection. All mice were housed in the South China Agricultural University Laboratory Animal Center. All procedures were received approval from the Ethics Committee of South China Agricultural University (2022d076). Pentobarbital sodium via intraperitoneal injection was used for both anaesthesia and euthanasia in this study.

### 2.3 Serum creatinine and irisin levels measurement

Blood samples were drawn and centrifuged at 700 *g* for 8 min to separate the serum. Serum creatinine and irisin concentrations were measured using a creatinine colorimetric assay kit (MEIMIAN) and an irisin ELISA kit (Biosharp).

### 2.4 Kidney histology and quantification

Kidney samples were preserved in 4% paraformaldehyde and then embedded in paraffin. Sections (4 µm) were stained with haematoxylin and eosin (HE) and periodic acid‐Schiff. Tubular damage was assessed based on loss of brush border, cellular necrosis or apoptosis and tubule dilation. For immunohistochemical staining, fixed and paraffin‐embedded tissue sections were incubated with an anti‐STING antibody (Cell Signalling Technology, 13647S, 1:200), followed by an incubation with optional secondary antibodies. Kidney sections were then counterstained with haematoxylin and prepared for microscopic evaluation.

### 2.5 Apoptosis analysis

Kidney tissue sections were analysed for apoptosis using a TUNEL assay kit. TUNEL‐positive cells were evaluated on a fluorescence microscope (Nikon) and counted in at least 10 areas per section, with three sections per kidney sample recorded.

### 2.6 Transmission electron microscopy

Fresh renal cortex tissue was harvested and pre‐fixed with 2% glutaraldehyde. The tissue was then fixed with 1% osmium tetroxide in the dark for 2 h. After dehydration with ethanol, the tissue was embedded using an acetone and epoxy resin mixture. Ultrathin sections (60‒80 nm) were cut and placed on copper grids. Subsequently, sections were stained with 2% uranium acetate‐saturated alcohol solution, and then observed using a Hitachi H‐7650 transmission electron microscope.

### 2.7 Western blot analysis

Tissues and cells were lysed using RIPA buffer. After protein concentration measurement, proteins (30 µg per sample) were resolved on 7.5%‒12.5% SDS‐PAGE gels and transferred onto polyvinylidene difluo‐ride (PVDF) membranes. Membranes were blocked and then incubated overnight with primary antibodies against cGAS, STING, TBK1, phospho‐TBK1, NF‐κB, phospho‐NF‐κB, Bax, VDAC1 and β‐actin (all from CST). The following day, secondary antibodies (Boster, 1:5000) were applied for 1 h. Protein bands were detected using a Tanon 5500 imaging system and analysed with ImageJ software.

### 2.8 Quantitative real‐time PCR

Total RNA was isolated using TRIzol reagent. The extracted RNA was then converted to cDNA using ReverTra Ace qPCR RT Master Mix (Takara). Quantitative PCR was conducted with SYBR Green PCR master mix (Takara). β‐Actin served as the internal control. The 2^−ΔΔCt^ method was used for quantitative analysis. Primer sequences for all analysed genes are listed in Table S3.

### 2.9 RNA sequencing

Kidney samples from WT saline, WT CI‐AKI, mPGC‐1α saline and mPGC‐1α CI‐AKI groups (*n* = 4/group) were collected. Total RNA was isolated, and its quality was evaluated using an Agilent 2100 Bioanalyser. cDNA libraries were prepared and sequenced on an Illumina Genome Analyser platform.

### 2.10 Cell culture and transfection

HK‐2 cell line was obtained from the American Type Culture Collection, cultured in Dulbecco's modified Eagle's medium (DMEM) supplemented with 10% foetal bovine serum (FBS). HK‐2 cells were treated with 40, 80 or 120 mg/mL of iohexol to assess responses. For cell transfection, HK‐2 cells were cultured in six‐well plates for 24 h, before being transfected with si‐STING or si‐cGAS (20 nM, GenePharma) using Lipofectamine 3000 transfection reagent (Thermo Scientific). Additionally, irisin treatment (500 ng/mL) was applied concurrently with iohexol stimulation to evaluate its effects.

### 2.11 Cell viability assay

Cells were plated in 96‐well plates (5000 cells/well) after iohexol treatment. Afterward, CCK‐8 solution (Beyotime, C0038) was added to each well and incubated for 4 h. The absorbance was then recorded at 450 nm.

### 2.12 Mitochondrial membrane potential

The mitochondrial membrane potential of HK‐2 cells was evaluated using a JC‐1 kit (Beyotime, C2006). Fluorescence was observed under a microscope, and the relative membrane potential was quantified by calculating the red‐to‐green fluorescence ratio.

### 2.13 Mito‐Tracker Red CMXRos assay

Cells were incubated with Mito‐Tracker dyes (200 nM, Beyotime, C1049B) at 37°C for 10 min, followed by Hoechst dyes (Beyotime, C1028) for 5 min. Mitochondrial morphology was then visualised using a Zeiss LSM880 microscope.

### 2.14 Immunofluorescence staining

Cells were fixed in 4% paraformaldehyde for 15 min. After fixation, cells were permeabilised using .5% Triton X‐100 for 15 min. Following a 1‐h blocking step at room temperature, the samples were incubated overnight with primary antibodies against TOMM20 (Abcam, ab186735) and dsDNA (Abcam, ab27156). Subsequently, the samples were incubated for 1 h at 37°C with secondary antibodies: goat anti‐rabbit immunoglobulin G (IgG) conjugated to either Alexa Fluor 594 (CST, 8889S) or Alexa Fluor 488 (CST, 4408S). After nuclei staining with DAPI, images were captured with a Zeiss LSM880 microscope.

### 2.15 Extraction and purification of cytosolic mtDNA

Cytosolic mtDNA extracts were prepared as previously detailed. HK‐2 cells were lysed with 100 µL of 1% NP‐40 lysate for 15 min. The insoluble fraction was precipitated by centrifugation at 16 000 *g* for 15 min. Supernatant (cytoplasmic fraction) was collected, and DNA was purified with a DNEasy Kit (QIAGEN, 69504). Cytosolic mtDNA levels were then quantified with a unique kit (Accurate, AG11701).

### 2.16 Co‐immunoprecipitation

HK‐2 cells were lysed with a modified RIPA buffer (Beyotime). For the Co‐immunoprecipitation assays, protein lysate was reserved as the input control. The experimental group included 40 µL of protein A/G‐agarose beads (Thermo Scientific), 960 µL of the protein lysate and 4 µg of either anti‐integrin αV (Abcam, ab179475) or anti‐integrin β5 (Abcam, ab309092) antibody. The IgG control group was also prepared, comprising 40 µL of beads, 960 µL of protein lysate and 4 µg of IgG. Then, samples were both incubated overnight at 4°C with gentle agitation. After performing five washes on the beads, the immunoprecipitated protein complex was resuspended in loading buffer and heated at 100°C for 10 min. Finally, our samples were subjected to SDS‐PAGE to resolve the immunoprecipitated proteins, facilitating further analysis.

### 2.17 Statistical analysis

Statistical analyses were conducted with SPSS 20.0 and GraphPad Prism 9.0. Differences among groups were evaluated with one‐way analysis of variance, followed by Tukey's post hoc test. Pearson correlation coefficient was applied to assess associations between plasma irisin levels and other variables. The significance was defined as *p*‐value <.05.

## 3 RESULTS

### 3.1 Low serum irisin as a predictive marker for CI‐AKI in cardiac procedure patients

**FIGURE 1:**
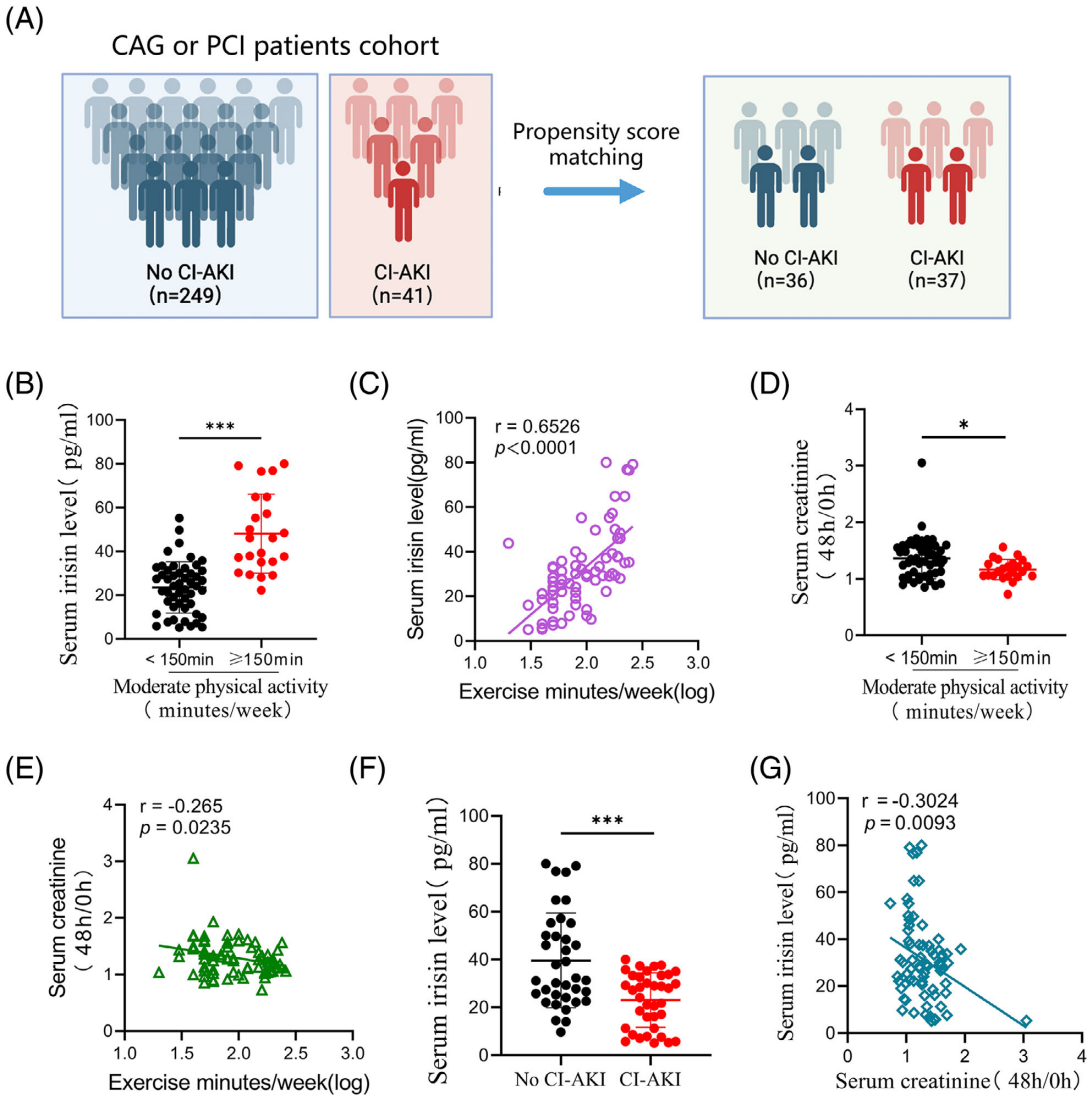
Low serum irisin as predictive marker for contrast‐induced acute kidney injury (CI‐AKI) in cardiac procedure patients. (A) Overview of the patient cohort for evaluating predictive value of irisin for CI‐AKI in patients undergoing cardiac procedures. (B) Participants were divided into two groups according to their exercise frequency, and then serum irisin levels were examined by ELISA assays (less than 150 min per week group, *n* = 50 and more than 150 min per week group, *n* = 23). (C) Pearson correlation coefficient between serum irisin levels and exercise minutes in participants described in (B) (*n* = 73). (D) The 48 h/0 h serum creatinine value of participants described in (B). (E) Linear regression analysis between the serum creatinine (48 h/0 h) and exercise minutes in individuals described in (B) (*n* = 73). (F) The serum irisin levels in the two groups of participants who were divided according to the occurrence of CI‐AKI (CI‐AKI group, *n* = 37 and no CI‐AKI group, *n* = 36). (G) Linear regression analysis between the serum irisin levels and serum creatinine (48 h/0 h) in individuals described in (B) (*n* = 73). Data are presented as means ± standard deviation (SD). Student's *t*‐test was used for statistical analysis. ^*^ *p* < .05; ^***^ *p* < .001.

To explore the co‐relationship between irisin levels in serum and CI‐AKI risk, we screened 290 patients undergoing CAG or PCI for inclusion (Figure S1). Notable differences in baseline characteristics were observed between the no CI‐AKI and CI‐AKI groups (Table S1). Our analysis plan included the selection of covariates based on previous studies, incorporating basic demographic characteristics (age, sex and body mass index) and factors relevant to CI‐AKI, such as estimated glomerular filtration rate, hypertension, diabetes mellitus, anaemia and chronic heart failure. Propensity score matching was conducted using a nearest‐neighbour 1:1 greedy matching technique within a caliper of .2 standard deviations of the logit of the propensity score.^23^ This process resulted in 36 patients with no CI‐AKI and 37 with CI‐AKI included in the analysis (Figure 1A). The predictive value of serum irisin was determined with receiver operating characteristic (ROC) curve. The analysis indicated irisin has a higher predictive value for CI‐AKI beyond cystatin C (CysC) and serum creatinine (Scr). The AUROC was determined to be .8475 (95% CI: .7862‒.9088, *p *< .0001) (Figure S1A). Current (2018) US guidelines for adults emphasised the importance of engaging in at least 150 min of moderate‐intensity physical activity each week to reduce the risks of non‐communicable diseases and premature mortality.^24^ Similarly, KDIGO guidelines recommend that individuals with CKD participate in moderate‐intensity exercise for a total of at least 150 min per week.^25^ Interestingly, patients who exercised frequently (moderate physical activity time more than 150 min per week, *n* = 23) had significantly higher serum irisin levels compared to those who exercised less frequently (moderate physical activity time less than 150 min per week, *n* = 50). The serum irisin concentrations were positively associated with exercise time per week (*r* = .6526, *p *< .0001, Figure 1B,C). Furthermore, the 48‐h/post‐PCI or CAG to baseline serum creatinine ratio was lower in the high exercise frequency group (Figure 1D,E). Importantly, serum irisin levels measured before PCI or CAG were significantly lower in the CI‐AKI group than in no CI‐AKI group (Figure 1F). Additionally, a negative correlation was observed between serum irisin levels and the 48‐h/baseline serum creatinine ratio (*r* = ‒.3024, *p *= .0093, Figure 1G), indicating that higher circulating irisin levels might be associated with a reduced risk of CI‐AKI. Thus, our findings indicated that higher serum irisin levels, particularly in patients who engage in frequent exercise, are associated with a lower risk of developing CI‐AKI post‐administration of contrast media. Univariate and multivariate logistic regression analyses were showed that irisin was associated with lower odds of CI‐AKI (Table S2). These results suggest that the risk of CI‐AKI could be linked to physical activity and the level of serum irisin might serve as a potential biomarker for predicting CI‐AKI in those patients undergoing cardiac procedures.

### 3.2 mPGC‐1α mitigates contrast‐induced acute kidney injury

**FIGURE 2:**
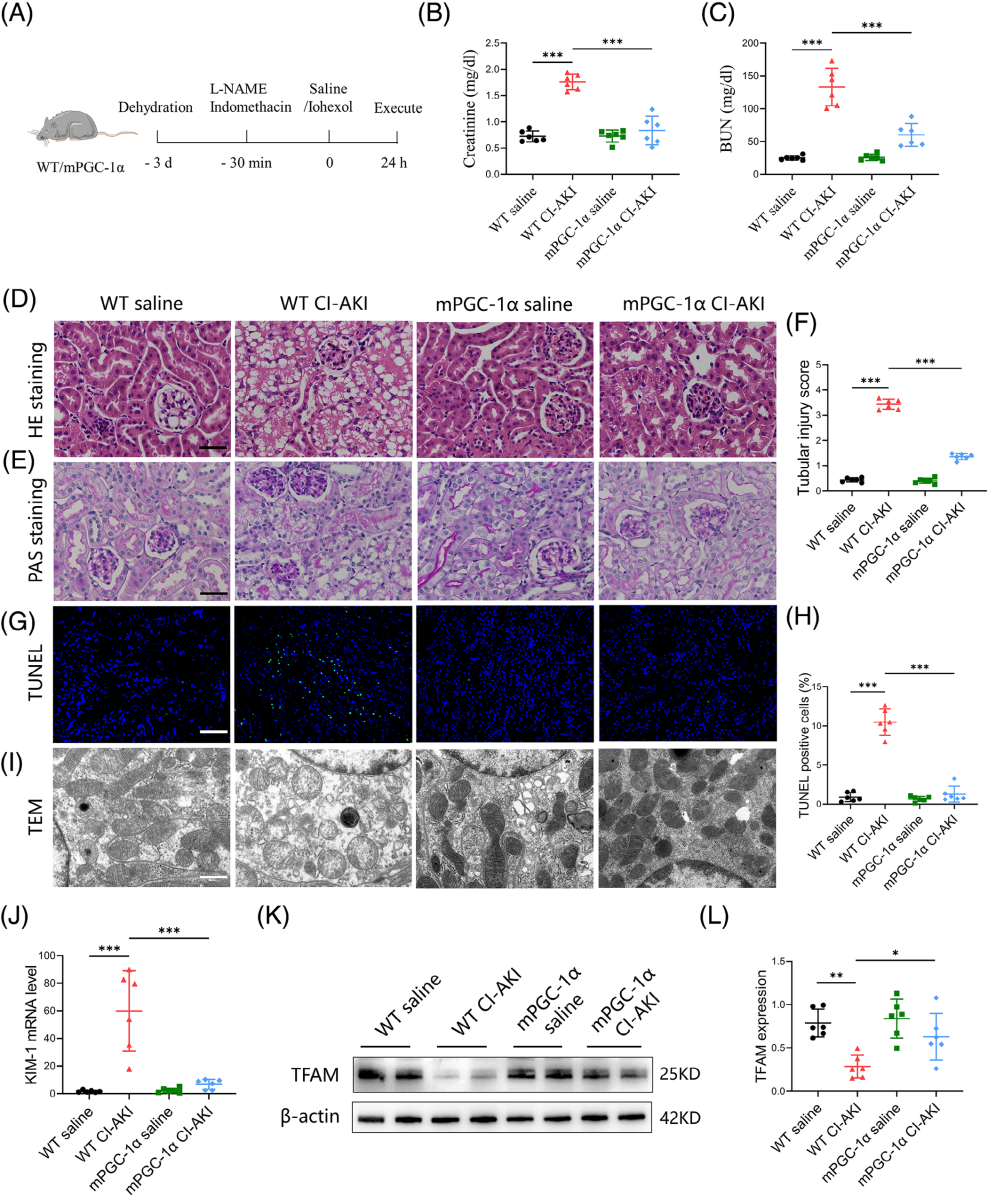
Muscle‐specific PGC‐1α (mPGC‐1α) mitigates contrast‐induced acute kidney injury. (A) Schematic diagram of the experimental design. Briefly, wild‐type (WT) and mPGC‐1α transgenic mice were treated with or without NG‐nitro‐L‐arginine methyl ester (L‐NAME; 10 mg/kg), indomethacin (10 mg/kg) and iohexol (5 gI/kg) after 3 days of dehydration. (B and C) Effects of mPGC‐1α on serum creatinine (B) and blood urea nitrogen (BUN) (C) levels (*n* = 6). (D and E) Representative images of haematoxylin and eosin (HE) staining (D) and periodic acid‐Schiff (PAS) staining (E) of renal cortex of WT saline, WT contrast‐induced acute kidney injury (CI‐AKI), mPGC‐1α saline and mPGC‐1α CI‐AKI mice. Scale bars: 100 µm. (F) The quantification of tubular injury based on the HE staining (*n* = 6). (G) Representative images of TUNEL staining of kidney sections. Scale bars: 200 µm. (H) The quantification of TUNEL‐positive cells in kidney tissues (*n* = 6). (I) Representative transmission electron microscopy (TEM) images of mitochondria in proximal tubule cells of mice. Scale bars: 1 µm. (J) The mRNA level of KIM‐1 of kidney tissues in in different groups as indicated (*n* = 6). (K and L) Western blotting analysis of TFAM in kidney tissues (*n* = 6). Data are presented as means ± standard deviation (SD). One‐way analysis of variance (ANOVA) with Tukey's post hoc test was used for statistical analysis. ^*^ *p* < .05; ^**^ *p* < .01; ^***^ *p* < .001.

To further investigate why high circulating irisin levels are associated with a lower occurrence of CI‐AKI, we utilised mPGC‐1α mice to simulate the effects of skeletal muscle exercise, leading to elevated circulating irisin levels. We developed a CI‐AKI mouse model by administering iohexol (5 gI/kg, once, ip) coupled with L‐NAME (10 mg/kg, once, ip) and indomethacin (10 mg/kg, once, ip, Figure S2A‒C).^26^ As depicted in Figure 2A, iohexol administration in WT mice led to a significant increase in serum creatinine levels, tubular injury scores, cast formation and intraepithelial vacuolar degeneration, confirming successful CI‐AKI modelling. In contrast, these indices of renal injury were markedly ameliorated in mPGC‐1α mice (Figure 2B‐F). To comprehensively assess the protective effects of mPGC‐1α on CI‐AKI, we employed the TUNEL assay to quantify apoptotic TECs and used a kidney injury molecule 1 (KIM‐1) antibody to detect injured renal tubules. The results, shown in Figure 2G,H,J, indicated a reduction in the proportion of TUNEL‐positive TECs in mPGC‐1α CI‐AKI mice, along with a downregulation of KIM‐1 mRNA levels compared to WT CI‐AKI mice. Transmission electron microscopy revealed that iohexol‐induced mitochondrial abnormalities, such as the disappearance of mitochondrial cristae, swelling and vacuolisation in TECs of WT CI‐AKI mice, were significantly reduced in mPGC‐1α CI‐AKI mice (Figure 2I). Furthermore, western blot analysis showed that mPGC‐1α reversed the decrease in transcription factor A, mitochondrial (TFAM), a protein essential for regulating mtDNA, observed in CI‐AKI mice (Figure 2K,L). Collectively, these data suggest that mPGC‐1α can attenuate CI‐AKI by preserving mitochondrial integrity and functions in TECs, reducing apoptosis, and mitigating renal tubular damage. This protective effect is likely mediated through the upregulation of irisin, which in turn positively influences mitochondrial function and cellular survival pathways within the kidneys.

### 3.3 mPGC‐1α inhibits activation of cGAS/STING axis and inflammation in injured kidney

**FIGURE 3:**
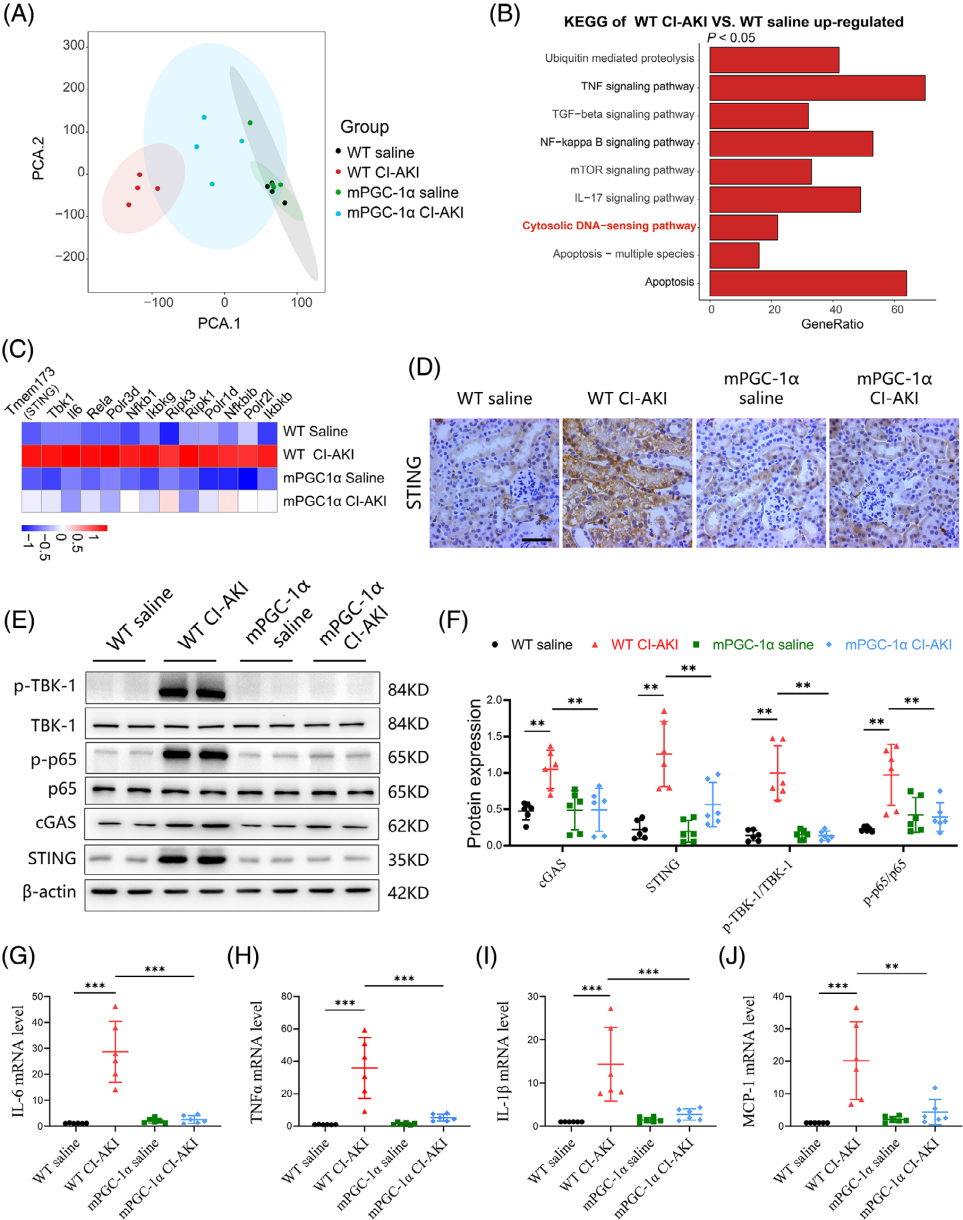
Muscle‐specific PGC‐1α (mPGC‐1α) inhibits activation of cGAS/STING axis and inflammation in injured kidney. (A) Principal component analysis (PCA) showing the marginal separation of the four groups (*n* = 4). (B) KEGG pathway analysis of upregulated genes in wild‐type (WT) contrast‐induced acute kidney injury (CI‐AKI) group indicated that cytosolic DNA‐sensing pathway was affected by iohexol treatment in kidney tissues (red mark). (C) Heatmap showing the relative expression of genes related to cytosolic DNA‐sensing pathway. (D) Representative STING staining images of kidney tissues in WT saline, WT CI‐AKI, mPGC‐1α saline and mPGC‐1α CI‐AKI groups. Scale bars: 100 µm. (E and F) Representative western blot images (E) and quantification analysis (F) of protein expression of cGAS, STING, p‐TBK1, TANK‐binding kinase 1 (TBK1), p‐p65 and p65 in kidney tissues (*n* = 6). (G‒J) Real‐time qPCR analysis showing the mRNA levels of MCP‐1, interleukin‐6 (IL‐6), tumour necrosis factor‐alpha (TNF‐α) and IL‐1β in kidney tissues (*n* = 6). Data are presented as means ± standard deviation (SD). One‐way analysis of variance (ANOVA) with Tukey's post hoc test was used for statistical analysis. ^**^ *p* < .01; ^***^ *p* < .001.

Given our findings that mPGC‐1α attenuates CI‐AKI, we next explored the potential underlying mechanisms. We performed RNA sequencing (RNA‐seq) analyses on kidney tissues from WT saline, WT CI‐AKI, mPGC‐1α saline and mPGC‐1α CI‐AKI mice. Principal component analysis indicated marginal separation between the WT saline and WT CI‐AKI groups based on mRNA abundance (Figure 3A). There were numerous differentially expressed genes in the kidneys of the WT saline and WT CI‐AKI mice (Figure S3A), while fewer gene changes were observed in the kidneys of mPGC‐1α CI‐AKI mice compared to uninjured mPGC‐1α saline mice (Figure S3B). Kyoto Encyclopedia of Genes and Genomes (KEGG) analysis revealed significant enrichment of the TNF and NF‐κB signalling pathways, as well as apoptosis, in the WT CI‐AKI group compared to the WT saline group, aligning with previous findings.^27^ Notably, the cytosolic DNA‐sensing pathway was also enriched in the WT CI‐AKI group (Figures 3B and S3C). Focusing on the genes of the cytosolic DNA‐sensing pathway, we found that mPGC‐1α repressed the activation of this pathway (Figure 3C). This was consistent with the differential expression of the STING (tmem173) gene observed in RNA‐seq data. Immunohistochemical staining showed that mPGC‐1α suppressed the increased STING expression in the renal tubulars of CI‐AKI mice (Figure 3D). Furthermore, we evaluated the activation of cGAS/STING axis by measuring the expression levels of cGAS and STING, and the phosphorylation of downstream molecules TBK1 and NF‐κB p65. mPGC‐1α significantly repressed cGAS/STING signalling activation induced by iohexol in kidney tissues (Figure 3E,F). Correspondingly, the mRNA levels of inflammation‐related cytokines were decreased in mPGC‐1α mice (Figure 3G‐J). In summary, our results reveal that mPGC‐1α significantly inhibits the cGAS/STING axis and reduces inflammation in CI‐AKI, underscoring the potential of targeting this pathway in CI‐AKI management.

### 3.4 cGAS‐STING activation mediates cell injury and inflammatory responses induced by iohexol

Building on our findings that mPGC‐1α inhibits the cGAS‐STING activation and reduces inflammation in CI‐AKI, we further investigated the specific mechanisms of iohexol‐induced renal injury at the cellular level. We examined its effects on tubular cell injury and mitochondrial dysfunction in vitro. HK‐2 cells treated with varying iohexol concentrations (0, 40, 80, 120 and 160 mgI/mL) for 12, 24 and 48 h exhibited dose‐dependent decreases in cell viability. Cell survival was reduced by 50% after 48 h of exposure to 40 mgI/mL iohexol, as determined by the CCK‐8 assay (Figure S4A). Iohexol treatment also induced mitochondrial dysfunction, as evidenced by changes in mitochondrial membrane potential (Figure S4B). Consistent with our findings in vivo, we observed the cGAS/STING axis activation in iohexol‐treated tubular cells. Western blot analysis demonstrated increased expression of STING and cGAS, accompanied by enhanced phosphorylation of downstream molecules TBK1 and NF‐κB p65 (Figure S4C,D). Crucially, knockdown of cGAS or STING significantly attenuated iohexol‐induced TBK1 and NF‐κB p65 phosphorylation (Figure S4E,F). These strong evidences uncover the key role of cGAS/STING signalling axis in mediating iohexol‐induced tubular cell injury.

### 3.5 STING inhibition mitigates renal injury and cytokine release in CI‐AKI mice

**FIGURE 4:**
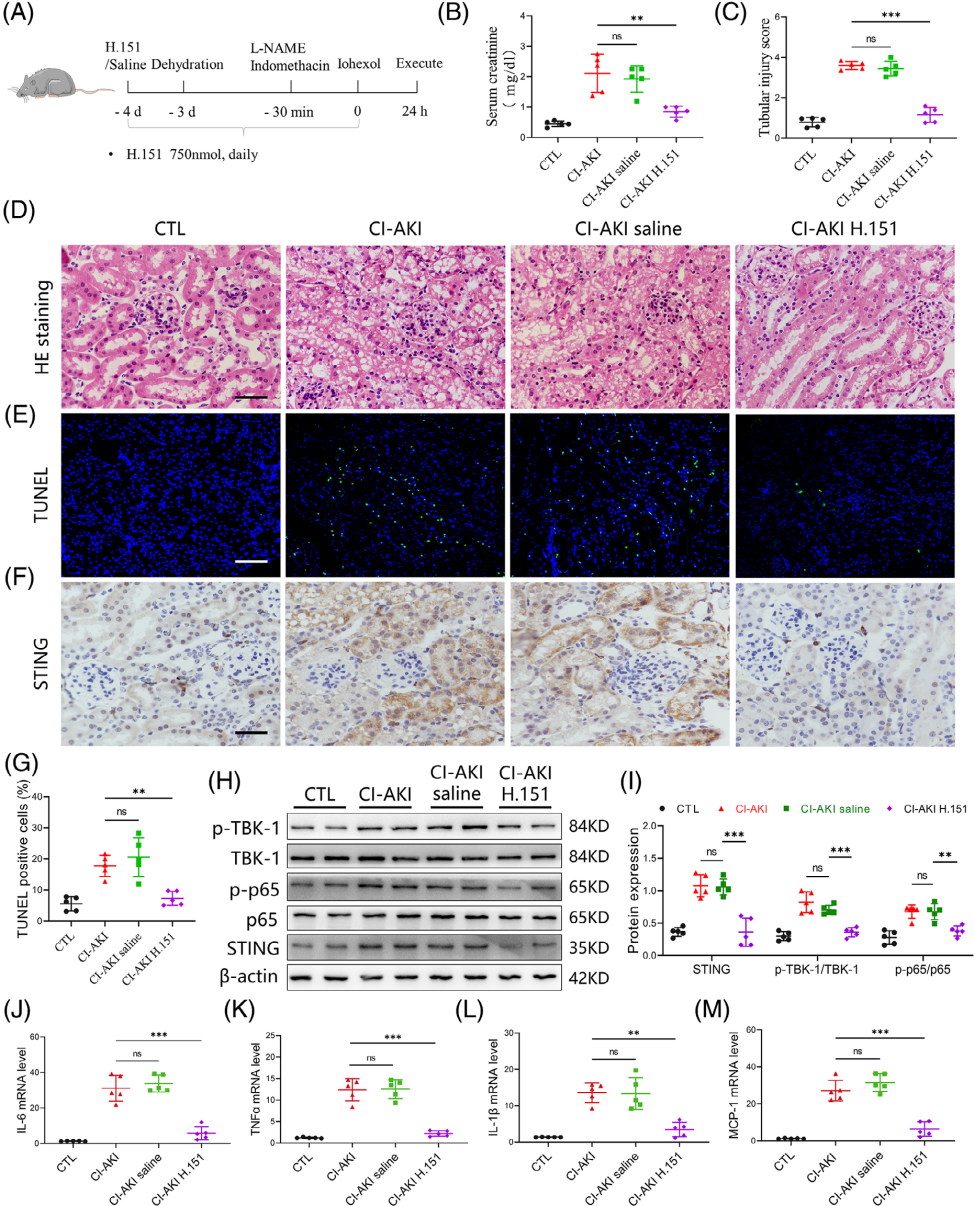
STING inhibition mitigates renal injury and cytokine release in contrast‐induced acute kidney injury (CI‐AKI) mice. (A) Schematic for the H.151 administration protocol. (B) Effects of H.151 on serum creatinine (*n* = 5). (C) The tubular injury score of control (CTL), CI‐AKI, CI‐AKI saline and CI‐AKI H.151 mice (*n* = 5). (D) Representative images of haematoxylin and eosin (HE) staining of groups described in (C) (*n* = 5). Scale bars: 100 µm. (E) TUNEL staining showing the cell apoptosis of kidney sections. Scale bars: 200 µm. (F) Representative STING staining views of kidney tissues of groups described in (C). Scale bars: 100 µm. (G) The quantification of TUNEL assay showing the reduction of apoptotic cells in kidney after H.151 treatment (*n* = 5). (H and I) The expression of cGAS, STING, p‐TBK1, TANK‐binding kinase 1 (TBK1), p‐p65 and p65 in kidney tissues were examined by western blot (*n* = 5). (J‒M) The mRNA levels of MCP‐1, interleukin‐6 (IL‐6), tumour necrosis factor‐alpha (TNF‐α) and IL‐1β in kidney tissues were examined by qPCR (*n* = 5). Data are presented as means ± standard deviation (SD). One‐way analysis of variance (ANOVA) with Tukey's post hoc test was used for statistical analysis. ^**^ *p* < .01; ^***^ *p* < .001; ns, no significance.

Having demonstrated the pivotal involvement of cGAS/STING axis in iohexol‐induced tubular cell injury in vitro, we next investigated the therapeutic potential of STING inhibition in a mouse model of CI‐AKI. CI‐AKI mice were pre‐treated with H.151, a small molecule antagonist of STING (750 nmol per mouse, daily, ip), 4 days before iohexol treatment to block STING activation.^28^ The experimental design and treatment regimen are illustrated in Figure 4A. We found that STING inhibition effectively reversed the increased serum creatinine levels and tubular injury scores observed in CI‐AKI mice (Figure 4B‐D). TUNEL staining further confirmed reduced cell apoptosis in the injured kidney due to STING inhibition (Figure 4E,G). Additionally, we assessed the activation of cGAS/STING signalling by western blot and immunohistochemistry. The results indicated that STING inhibition downregulated the expression of STING and decreased the phosphorylation levels of TBK1 and NF‐κB p65 in the injured kidney (Figure 4F,H,I). Furthermore, the release of cytokines, including IL‐1β, monocyte chemoattractant protein‐1 (MCP‐1), IL‐6 and TNF‐α was suppressed by STING inhibition (Figure 4J‒M). Collectively, these in vivo results strongly reinforce the pivotal role of cGAS/STING signalling in CI‐AKI development and highlight the therapeutic potential of STING inhibition in reducing renal injury and inflammation.

### 3.6 Irisin alleviates CI‐AKI by inhibiting cGAS‐STING activation

**FIGURE 5:**
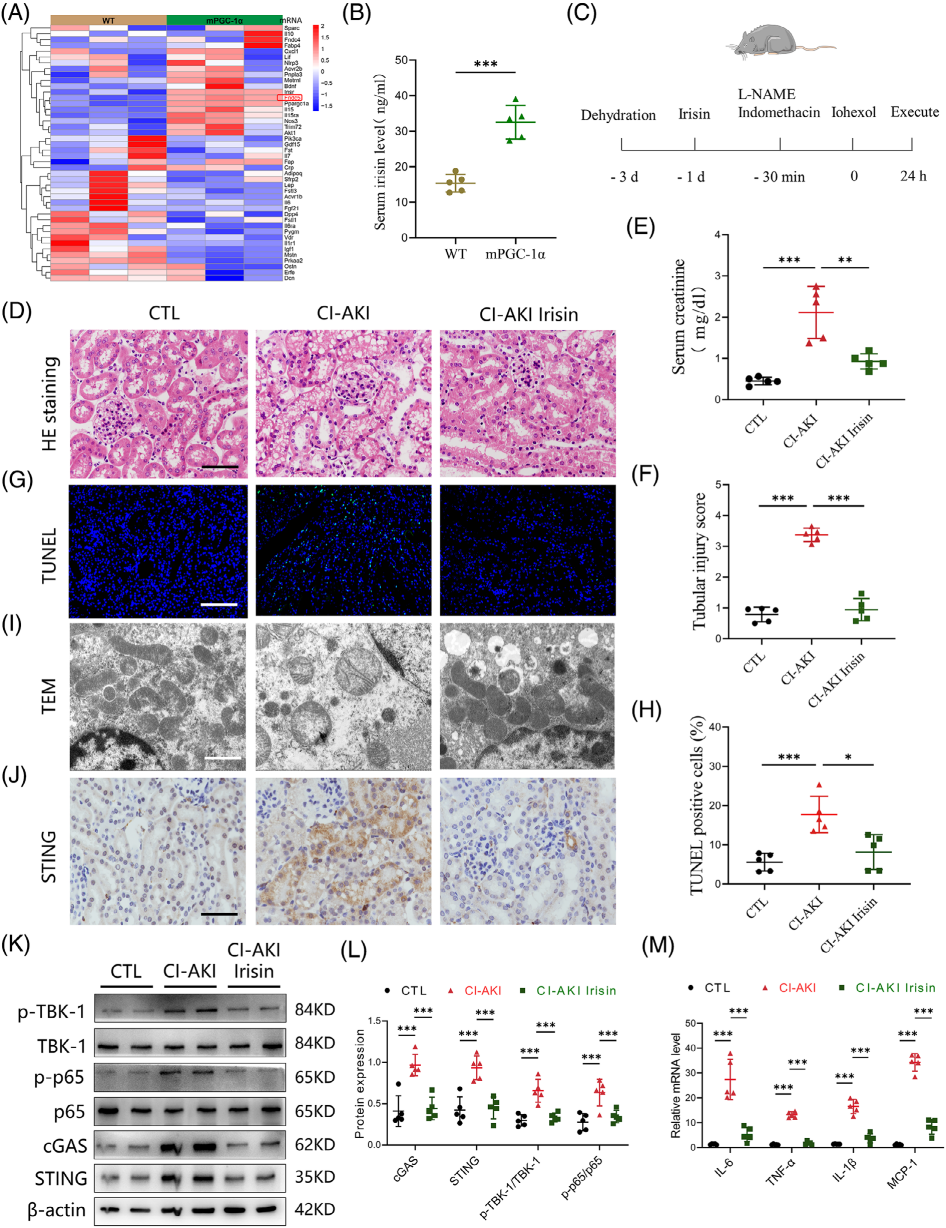
Irisin alleviates contrast‐induced acute kidney injury (CI‐AKI) by inhibiting cGAS‐STING activation. (A) Heatmap showing the differential expression of myokines in the tibialis anterior muscles between wild‐type (WT) mice and muscle‐specific PGC‐1α (mPGC‐1α) mice. Irisin/Fndc5 was one of the most upregulated myokines in mPGC‐1α mice (*n* = 3). (B) Serum concentration of irisin in WT and mPGC‐1α mice (*n* = 3). (C) Schematic diagram of the experimental design. Briefly, irisin (200 µg/kg) was injected into mice 1 day before iohexol treatment. (D) Representative images of haematoxylin and eosin (HE) staining of CTL, CI‐AKI and CI‐AKI irisin groups. Scale bars: 100 µm. (E) The serum creatinine levels of the three groups (*n* = 5). (F) Tubular injury score of the three groups (*n* = 5). (G and H) TUNEL assay showed reduced apoptosis in tubules from CI‐AKI mice with irisin (*n* = 5). Scale bars: 200 µm. (I) Transmission electron microscopy (TEM) images revealed protection in mitochondria injury in tubules from CI‐AKI mice with irisin. Scale bars: 1 µm. (J‒L) STING staining and western blot analysis of proteins related to cGAS‐STING signalling show that iohexol‐induced activation of cGAS‐STING signalling was inhibited by irisin treatment (*n* = 5). Scale bars: 100 µm. (M) Relative mRNA levels of interleukin‐6 (IL‐6), MCP‐1, tumour necrosis factor‐alpha (TNF‐α) and IL‐1β in kidney tissues were examined by real‐time qPCR (*n* = 5). Data are presented as means ± standard deviation (SD). Student's *t*‐test and one‐way analysis of variance (ANOVA) with Tukey's post hoc test were used for statistical analysis. ^*^ *p* < .05; ^**^ *p* < .01; ^***^ *p* < .001.

Building on our findings implicating the cGAS‐STING pathway in CI‐AKI, we hypothesised that certain key myokines played a central role in alleviating CI‐AKI. To test the hypothesis, we analysed the mRNA expression levels of myokines in the muscles of WT and mPGC‐1α mice used a PCR‐based array to. As shown in Figure 5A, several myokine‐coding genes, such as Bdnf, Fndc5, Insr, Il15 and Nos3, were upregulated in mPGC‐1α mice. We also confirmed the serum level of irisin was higher in mPGC‐1α mice than WT mice (Figure 5B). Focusing on FNDC5/irisin, which has been suggested to mediate muscle‐renal crosstalk and delay the progression of CKD, we investigated whether irisin released by muscles could alleviate renal injury induced by iohexol. Mice were pre‐treated with recombinant irisin (200 µg/kg, once, ip) one day before iohexol administration (Figure 5C), because 200 µg/kg dose treatment was more effective in protecting kidney injury than those of 100 or 400 µg/kg doses (Figure S5A‐C). Next, mouse serum and kidney tissues were collected for creatinine measurement, HE and TUNEL staining. The results showed that irisin treatment reduced renal injury and serum creatinine levels, alongside a decrease in apoptotic cells (Figure 5D‐H). Notably, irisin also reversed mitochondrial injury in TECs of CI‐AKI mice (Figure 5I). To determine if Irisin mediates the repression of the cGAS‐STING signalling in the kidneys of mPGC‐1α mice, we analysed the expression levels of STING and cGAS, along with the phosphorylation status of TBK1 and NF‐κB p65. The findings confirmed that irisin treatment repressed the cGAS‐STING signalling (Figure 5J‐L). Consistent with these observations, the release of inflammatory factors was also reduced (Figure 5M). These results demonstrate that irisin, a myokine upregulated in mPGC‐1α mice, plays a crucial role in reducing renal injury and inflammation response in CI‐AKI, acting through cGAS/STING repression and protection of mitochondrial integrity in TECs.

### 3.7 Irisin ameliorates iohexol‐induced mitochondrial impairment and mtDNA release

**FIGURE 6:**
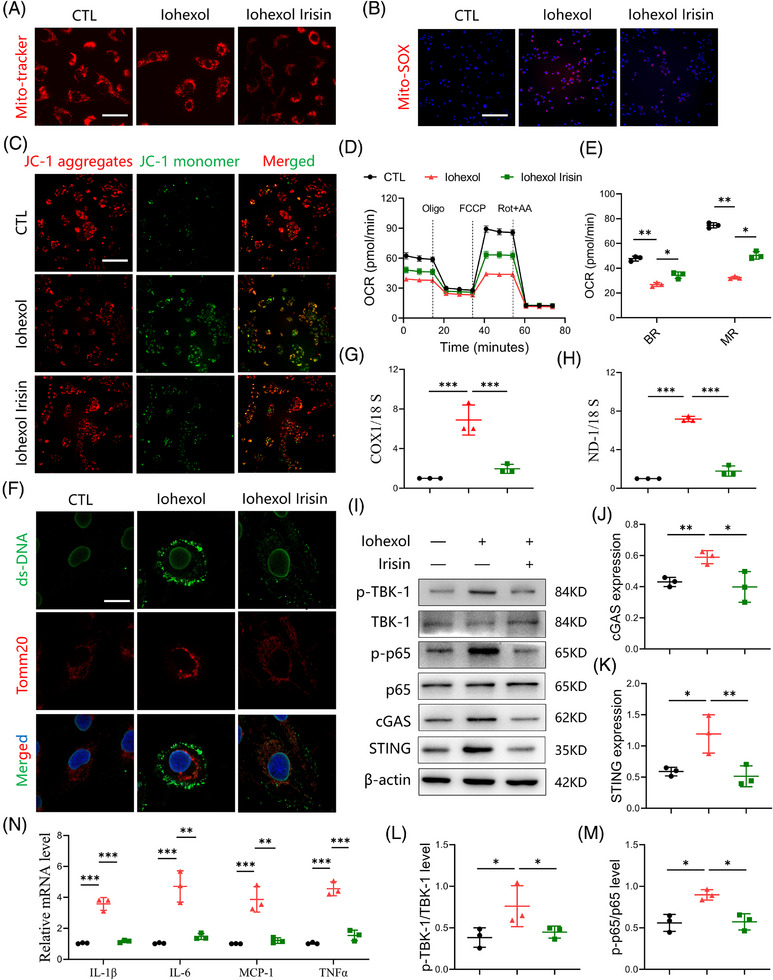
Irisin ameliorates iohexol‐induced mitochondrial impairment and mitochondrial DNA (mtDNA) release in tubular epithelial cells (TECs). (A) Representative mitochondrial micrographs stained by Mito‐Tracker (red) in TECs. Scale bar: 100 µm. (B) Representative images show mitochondrial reactive oxygen species (mt‐ROS) levels in the TECs from different groups. Scale bar: 200 µm. (B) Immunofluorescence of the mitochondrial membrane potential in TECs labelled with JC‐1. Scale bar: 200 µm. (D and E) Real‐time changes in the oxygen consumption rate (OCR) of TECs were detected by Seahorse assay (*n* = 3). BR, basal respiration; MR, maximal respiration. (F) The co‐staining of dsDNA (green) and Tomm20 (red) showed a decrease in the leakage of dsDNA after irisin treatment. DAPI: blue. Scale bar: 20 µm. (G and H) The mRNA levels of COX1/18S (G) and ND‐1/18S (H) in mitochondria‐free cytosolic fractions were detected by real‐time qPCR (*n* = 3). (I‒M) Western blot analysis showing the expression of proteins related to cGAS‐STING signalling in TECs (*n* = 3). (N) Real‐time qPCR assay showing the relative mRNA levels of interleukin‐1β (IL‐1β), IL‐6, MCP‐1 and tumour necrosis factor‐alpha (TNF‐α) in TECs (*n* = 3). Data are presented as means ± standard deviation (SD). One‐way analysis of variance (ANOVA) with Tukey's post hoc test was used for statistical analysis. ^*^ *p* < .05; ^**^ *p* < .01; ^***^ *p* < .001.

Building upon our established findings, our next objective was to clarify the impact of irisin on mitochondrial function within TECs subjected to iohexol exposure. As shown in Figure 6A‐E, irisin exposure led to notable reductions in mitochondrial dysfunction after iohexol stimulation, as determined by the preservation of mitochondrial structural integrity, reduction in mitochondrial reactive oxygen species (ROS) level, and increase in the mitochondrial membrane potential level, oxygen consumption rate. Notably, we observed that double stranded DNA (dsDNA) was released into the cytoplasm in iohexol‐stimulated HK‐2 cells, potentially contributing to cGAS‐STING signalling activation. Treatment with irisin (500 ng/mL) partially reduced this dsDNA leakage (Figure 6F). Furthermore, we isolated mitochondria‐free cytosolic fractions from HK‐2 cells and conducted a PCR assay to quantify mtDNA. The results indicated that both COX1/18S and ND‐1/18S ratios were reduced following irisin treatment (Figure 6G,H). It is reported that the translocation of Bax from cytosol to mitochondria induces the formation of Bax macropores, increasing the permeability of mitochondrial membrane and promotes the release of mtDNA. We then identified that iohexol exposure enhanced the expression of Bax in mitochondria while downregulated the expression of Bax in cytosol. Notably, the transfer of Bax from cytosol to the mitochondria was greatly suppressed by irisin treatment (Figure S6A,B).

The leakage of dsDNA can activate the cGAS‐STING signalling in TECs. To confirm that irisin inhibits this activation by preventing dsDNA leakage, we assessed the levels of cGAS/STING‐related proteins and downstream inflammatory factors. As expected, irisin treatment decreased the expression of c‐GAS and STING proteins, as well as the phosphorylation of NF‐κB p65 and TBK1 in iohexol‐stimulated HK‐2 cells. This was accompanied by reduced levels of several inflammatory factors (Figure 6I‐N). Collectively, these findings demonstrate that irisin alleviates iohexol‐induced mitochondrial damage in TECs and prevents mtDNA release. This inhibition of cGAS/STING pathway and subsequent decrease in inflammation underscore the protective role of irisin in mitochondrial integrity and offer a potential therapeutic strategy for CI‐AKI.

### 3.8 Integrin complex αV/β5 receptor in TECs is required for irisin‐induced inhibition of cGAS‐STING pathway

**FIGURE 7:**
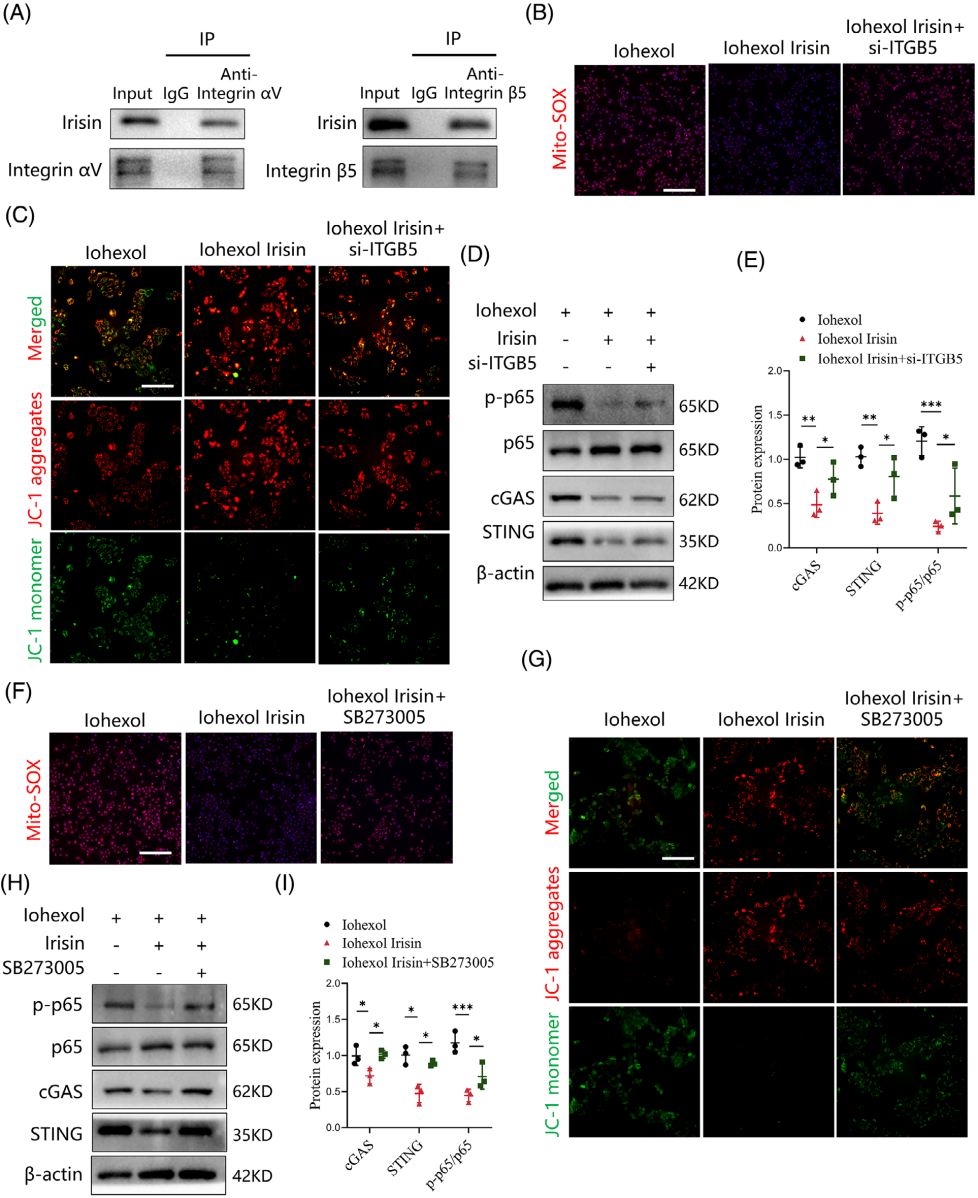
Integrin αV/β5 receptor in tubular epithelial cells (TECs) is required for irisin‐induced inhibition of cGAS‐STING pathway. (A) Representative immunoblots of irisin/FNDC5, integrin αV and integrin β5/ITGB5 in a co‐immunoprecipitation assay of TECs treated with irisin. Immunoglobulin G (IgG) indicates the control antibody group. (B) Representative image of Mito‐SOX red staining in the TECs from different groups. Scale bar: 200 µm. (C) Immunofluorescence of the mitochondrial membrane potential in TECs labelled with JC‐1. Scale bar: 200 µm. (D and E) Western blot analysis showing the expression of proteins related to cGAS‐STING signalling in TECs (*n* = 3). (F) Representative image of Mito‐SOX red staining in TECs. Scale bar: 200 µm. (G) Immunofluorescence of the mitochondrial membrane potential in TECs. Scale bar: 200 µm. (H and I) Protein expressions of cGAS, STING, p65 and phosphorylation of p65 in TECs were examined by western blot (*n* = 3). Data are presented as means ± standard deviation (SD). Student's *t*‐test and one‐way analysis of variance (ANOVA) with Tukey's post hoc test were used for statistical analysis. ^*^ *p* < .05; ^**^ *p* < .01; ^***^ *p* < .001.

Previous studies identified the integrin αV/β5 as a receptor for irisin in various cell types, including osteocytes, myocardial cells and adipocytes.^18^, ^29^ The integrin αV subunit exclusively pairs with integrin β5.^30^ In this study, we confirmed the presence of integrin αV and integrin β5 proteins in TECs. However, their expression remained unaltered following iohexol stimulation (Figure S7A,B). Next, we sought to investigate whether integrin αV/β5 receptor is essential for irisin's protective effects on mitochondrial function and suppression of the cGAS‐STING pathway. Co‐immunoprecipitation assays demonstrated that irisin/FNDC5 physically interacted with integrin αV and integrin β5/ITGB5 in TECs treated with recombinant irisin (Figure 7A). We then generated small interfering RNA to block integrin β5/ITGB5 activity genetically, and found that irisin lost its abilities to reduce mitochondrial ROS generation, increase mitochondrial membrane potential level, and inhibit the cGAS‐STING pathway (Figure 7B‐E). To block integrin αV/β5 activity pharmacologically, TECs were incubated with SB273005, an integrin αV/β5 inhibitor. The treatment of SB273005 negated the beneficial effects of irisin on mitochondrial dysfunction, as determined by the reduction in mitochondrial ROS production, and increase in mitochondrial membrane potential level (Figure 7F,G). Moreover, SB273005 effectively reversed the suppression of cGAS‐STING signalling after irisin stimulation by detecting the levels of cGAS, STING and p‐p65 (Figure 7H,I). These findings highlight integrin αV/β5 as a special receptor for irisin in TECs, mediating its protective effects by alleviating mitochondrial dysfunction and suppressing cGAS‐STING signalling.

## 4 DISCUSSION

CI‐AKI is a significant challenge in the clinical management of patients with coronary artery disease undergoing imaging or therapeutic procedures. It is caused by using contrast agents, which can lead to a rapid deterioration in kidney function.^27^ Despite its recognised impact on patient outcomes and healthcare systems, effective preventive and mitigating strategies remain elusive. To address this challenge, our study utilised the mPGC‐1α transgenic mouse model, an innovative surrogate for exercise‐induced physiological responses, to explore the therapeutic potential of exercise mimetics in this context.^15^ After a comprehensive evaluation, we found that mPGC‐1α overexpression exerted significant protective effects against contrast‐induced kidney injury. Specifically, mPGC‐1α transgenic mice exhibited markedly reduced apoptosis, maintained mitochondrial structure and function, and attenuated inflammatory response in TECs. These data underscore the pivotal role of ‘muscle‒kidney crosstalk’ in protecting renal function from the detrimental effects of contrast agent exposure.

By elucidating the potential mechanisms underlying this protection, our results highlight the renoprotective functions of myokines, particularly irisin: its ability to alleviate mitochondrial dysfunction, reduce the secretion of pro‐inflammatory cytokines, and suppress cGAS/STING activation in TECs. These findings advance our understanding of the potential mechanisms of ‘muscle‒kidney crosstalk’. While the benefits of exercise in promoting overall health are well‐established, its implementation as a preventive or therapeutic measure for CI‐AKI is not practical for patients with acute coronary syndrome. Therefore, our study lays the foundation for exploring exercise mimetics as a novel therapeutic avenue for CI‐AKI.

Mitochondrial dysfunction stands at the forefront of pathophysiological processes leading to renal injury, particularly in AKI.^31^, ^32^, ^33^ The vulnerability of TECs to disruptions in mitochondrial homeostasis underscores the critical importance of mitochondrial integrity for renal function. This dysfunction, characterised by impaired ATP production, increased generation of ROS, and altered mitochondrial dynamics, triggers a cascade of cellular events leading to TEC injury and apoptosis. Intriguingly, the cGAS‐STING signalling pathway is intimately associated with mitochondrial dysfunction due to its role in detecting mtDNA that leaks into the cytoplasm under mitochondrial stress or damage.^34^ Recent research has underscored the pivotal role of cGAS‐STING pathway in renal diseases, highlighting its promise as a potential target for therapeutic intervention.^35^, ^36^ Activating this pathway in response to cellular stress and DNA damage can exacerbate renal diseases by inducing inflammation. Conversely, controlled modulation of this pathway represents a promising strategy for mitigating renal injury by reducing inflammatory response. Our findings, which show the effectiveness of irisin or H.151 treatment in reversing mitochondrial damage and inhibiting cGAS‐STING pathway, support this therapeutic approach.

Irisin emerged as a focal point in our study because it is an exercise‐induced myokine that regulates metabolic functions across various organs, including the kidneys. Although its effects transcend the conventional scope of muscle tissue, the precise mechanisms by which it operates have yet to be discovered. Our research illuminated the involvement of irisin in modulating the cGAS‐STING signalling, albeit not through direct interaction. Instead, our data revealed that irisin administration preserves the mitochondrial integrity of TECs exposed to iohexol, a widely used contrast agent. This preservation prevents mtDNA from leaking into the cytosol, thereby inhibiting cGAS/STING axis activation and reducing expression of inflammatory cytokines and markers of cellular injury. These outcomes underscore the capacity of irisin to counteract the detrimental impacts of contrast agents by deploying a comprehensive defense strategy that encompasses mitochondrial protective, antioxidant and anti‐inflammatory actions. Thus, the interplay between mitochondrial integrity, the cGAS‐STING signalling pathway, and CI‐AKI development elucidate a complex mechanism of renal injury modulated by exercise‐induced myokines such as irisin.

Given that each myokine exerts its effects through unique receptor interactions and signal transduction cascades, these interactions may be crucial for protecting the kidneys and maintaining systemic equilibrium. Therefore, identifying and characterising these myokines and their mechanisms of action are critical steps in fully understanding the communication mechanisms between muscles and kidneys. We confirmed integrin complex αV/β5 as the specific receptor through which irisin interacts with TECs.

While our study has uncovered several key findings, it also has some limitations. Our study has yet to rule out the possibility that other myokines might be involved in this protective process, which points to directions for future research.

**FIGURE 8:**
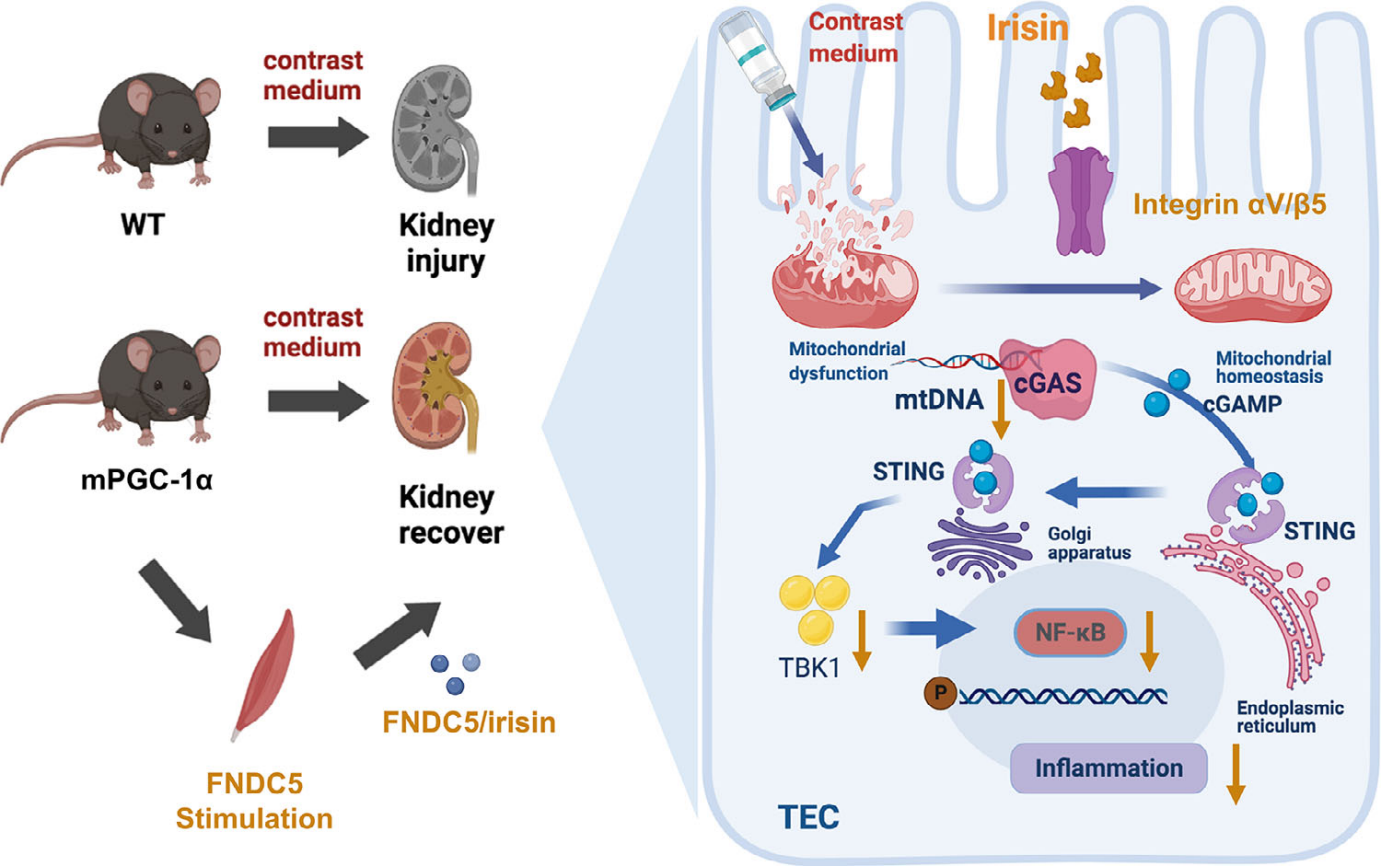
Schema depicting regulations of irisin released by muscle‐specific PGC‐1α (mPGC‐1α) on cGAS‐STING pathway in tubular epithelial cells (TECs).

In summary, our study demonstrates that mPGC‐1α plays a pivotal role in alleviating CI‐AKI by promoting irisin secretion, enhancing muscle‒kidney crosstalk, and mitigating mitochondrial damage, thereby inhibiting the cGAS‐STING signalling pathway and inflammation (Figure 8). These new findings not only deepen our understanding of the muscle‒kidney interaction in CI‐AKI but also lay the foundation for the potential clinical application of irisin in CI‐AKI treatment.

## AUTHOR CONTRIBUTIONS

Hui Peng initiated and designed this study. Hui Peng, Zhaoyong Hu and Weiyan Lai supervised this study. Long Peng, Suhua Li and Qiang Huang performed most of the molecular biological experiments and animal experiments. Yuxiang Sun and Juan Sun assisted with animal experiments. Ting Luo and Yanlin Wang provided professional guidance and technical support. Long Peng and Qiang Huang analysed the data. Long Peng and Qiang Huang wrote the manuscript. Hui Peng, Suhua Li and Zhaoyong Hu edited and reviewed the manuscript.

## CONFLICT OF INTEREST STATEMENT

The authors declare they have no conflicts of interest.

## ETHICS STATEMENT

The research protocol received approval from the Ethics Committee of the Third Affiliated Hospital of Sun Yat‐Sen University (2022‐02‐194‐01), and written informed consent was obtained from all participants.

## Supporting information



Supporting Information

## Data Availability

The data supporting the findings of this study can be made available by the corresponding author (Hui Peng) upon reasonable request.
