# Supplementary material for: Irisin‐mediated muscle‐renal crosstalk as a protective mechanism against contrast‐induced acute kidney injury via cGAS‐STING signalling inhibition
Source: Clin Transl Med. 2025 Feb 26;15(3):e70235. doi: 10.1002/ctm2.70235 (PMC11862893; doi:10.1002/ctm2.70235)
Supplement: Supplementary file 1 — Supporting Information [file CTM2-15-e70235-s001.docx]

**Irisin mediated muscle-renal crosstalk as a protective mechanism against contrast induced acute kidney injury via cGAS-STING signaling inhibition**

Long Peng^1,2#^, Suhua Li^1#^, Qiang Huang^2#^, Yuxiang Sun^2#^, Juan Sun^2^, Ting Luo^2^, Yanlin Wang^3^, Zhaoyong Hu^4^, Weiyan Lai^2*^, Hui Peng^2*^

**Table S1. Baseline Characteristics of No CI-AKI and CI-AKI Patients.**

|  | Unmatched original data set | | | Propensity score—matched data set | | |
| --- | --- | --- | --- | --- | --- | --- |
|  | No CI-AKI  （n=249） | CI-AKI  （n=41） | *p* Value | No CI-AKI  （n=36） | CI-AKI  （n=37） | *p* Value |
| Age, years | 63.89±10.66 | 63.46±11.50 | 0.81 | 63.72±9.52 | 62.86±11.89 | 0.735 |
| Male, n (%) | 197（79.1%） | 26（63.4%） | 0.027 | 25(69.4%) | 22(59.5%) | 0.373 |
| BMI, kg/m^2^ | 24.53±4.06 | 23.95±4.22 | 0.397 | 23.9±3.09 | 24.07±4.34 | 0.849 |
| Systolic blood pressure, mmHg | 133.82±20.25 | 140.29±23.87 | 0.06 | 137.61±20.51 | 140±23.74 | 0.578 |
| Diastolic blood pressure, mmHg | 81.36±12.16 | 82.59±20.53 | 0.595 | 81.19±9.47 | 82.41±20.41 | 0.747 |
| Heart rate, bpm | 77.24±12.51 | 85.26±16.63 | 0.000 | 77.06±12.36 | 82.92±14.97 | 0.145 |
| Hypertension, n (%) | 155（62.2%） | 29（70.7%） | 0.296 | 21(58.3%) | 25(67.6%) | 0.414 |
| Diabetes mellitus, n (%) | 97（39%） | 18（43.9%） | 0.549 | 18(50%) | 14(37.8%) | 0.295 |
| Hyperlipidemia, n (%) | 77（30.9%） | 14（34.1%） | 0.680 | 15(41.7%) | 13(35.1%) | 0.566 |
| CHF, n (%) | 10（4.0%） | 11（26.8%） | 0.000 | 6(16.7%) | 9(24.3%) | 0.418 |
| Anemia, n (%) | 51(20.5%) | 13(31.7%) | 0.108 | 6(16.7%) | 9(24.3%) | 0.418 |
| Current smoker, n (%) | 100（40.2%） | 17（41.5%） | 0.875 | 10(27.8%) | 16(43.2%) | 0.168 |
| Hemoglobin, g/L | 136.8±17.74 | 130.76±18.95 | 0.046 | 133.92±19.65 | 133.68±17.41 | 0.956 |
| Total cholesterol, mM | 4.41±1.34 | 4.70±1.31 | 0.196 | 4.47±1.46 | 4.65±1.32 | 0.566 |
| Triglyceride, mM | 1.83±1.75 | 1.87±1.74 | 0.885 | 1.42±1.46 | 4.65±1.32 | 0.191 |
| HDL-C, mM | 0.98±0.28 | 0.93±0.22 | 0.321 | 1.05±0.31 | 1.85±1.79 | 0.132 |
| LDL-C, mM | 2.64±1.05 | 3.02±1.21 | 0.038 | 2.70±1.16 | 2.99±1.24 | 0.296 |
| Fasting glucose, mM | 6.39±2.56 | 7.57±4.15 | 0.082 | 6.21±1.63 | 6.93±2.56 | 0.157 |
| HbA1C, % | 6.81±5.04 | 6.62±1.51 | 0.821 | 6.93±1.36 | 6.63±1.58 | 0.405 |
| BUN, μM | 7.80±24.45 | 14.74±46.79 | 0.151 | 6.09±2.14 | 6.39±2.20 | 0.553 |
| Scr, μM | 76.41±28.22 | 121.12±128.20 | 0.000 | 72.24±12.36 | 82.92±14.97 | 0.174 |
| eGFR, mL/min/1.73 m2 | 87.88±19.83 | 74.23±33.74 | 0.000 | 88.75±21.11 | 81.01±27.89 | 0.186 |
| LVEF, % | 64.28±8.01 | 60.17±9.53 | 0.003 | 63.00±9.79 | 60.78±9.74 | 0.336 |
| ACEI/ARB/ARNI, n (%) | 110(44.2%) | 27(65.9%) | 0.01 | 16(44.4%) | 22(59.5%) | 0.199 |
| Statins, n (%) | 239（96%） | 37（90.2%） | 0.112 | 33(91.6%) | 33(89.2%) | 0.719 |
| Diuretic, n (%) | 37（14.9%） | 14（34.1%） | 0.003 | 5(13.8%) | 10(27.0%) | 0.165 |
| Procedure, n (%) |  |  | 0.509 |  |  | 0.741 |
| CAG | 86（87.8%） | 12（12.2%） |  | 13(52%) | 12(48%) |  |
| PCI | 163（84.9%） | 29（15.1%） |  | 23(47.9%) | 25(52.1%) |  |
| Contrast volume, mL | 133.24±66.41 | 147.07±76.82 | 0.228 | 136.94±71.98 | 144.86±78.62 | 0.655 |
| Irisn （pg/ml） | 43.97±15.01 | 23.66±13.77 | 0.000 | 41.88±20.36 | 23.33±11.92 | 0.000 |
| Cystatin C（mg/L） | 3.285±1.605 | 4.821±2.989 | 0.7108 | 4.739±3.494 | 4.729±3.314 | 0.9983 |

Means ± SD for continuous variables, *P* < 0.05 presents significant difference. BMI: Body Mass Index; CHF: Congestive Hearts Failure; HDL-C: High Density Lipoprotein Cholesterol; LDL-C: Low Density Lipoprotein Cholesterol; BUN: Blood Urea Nitrogen; Scr: Serum Creatinine; eGFR: Estimated Glomerular Filtration Rate; LVEF: Left Ventricular Ejection Fraction; ACEI/ARB/ARNI: Angiotensin Converting Enzyme Inhibitor (ACEI)/Angiotensin Receptor Blocker (ARB)/Angiotensin Receptor Neprilysin Inhibitor (ARNI); CAG: Coronary Angiography; PCI: Percutaneous Coronary Intervention.

**Table S2. Univariate and multivariate logistic regression analysis of the association between irisin and CI-AKI (No CI-AKI, n=249 and CI-AKI, n=41).**

|  | Model 1 | |  | Model 2 | |  | Model 3 | |
| --- | --- | --- | --- | --- | --- | --- | --- | --- |
|  | OR(95%CI) | P value |  | OR(95%CI) | P value |  | OR(95%CI) | P value |
| Irisin T1 | 1.00(Reference) | - |  | 1.00(Reference) | - |  | 1.00(Reference) | - |
| Irisin T2 | 0.16(0.07-0.38) | <0.001 |  | 0.15(0.06-0.37) | <0.001 |  | 0.16(0.05-0.45) | 0.001 |
| Irisin T3 | 0.04(0.01-0.18) | <0.001 |  | 0.04(0.01-0.16) | <0.001 |  | 0.03(0.01-0.16) | <0.001 |
| Irisin per 1-SD | 0.21(0.13-0.34) | <0.001 |  | 0.19(0.11-0.32) | <0.001 |  | 0.12(0.05-0.26) | <0.001 |

Model 1 was univariate logistic regression analysis. Model 2 was adjusted for age, gender and BMI. Model 3 was further adjusted for hypertension, diabetes, smoking, heart failure, CKD, eGFR, HGB, TC, TG, FPG, HbA1c, LVEF, drug, procedure, contrast volume.

**Table S3. The primers of quantitative PCR.**

| Primer Type |  |  |  |
| --- | --- | --- | --- |
| TNFα | mouse | Forward | CGCTCTTCTGTCTACTGAACTTCGG |
|  |  | Reverse | GTGGTTTGTGAGTGTGAGGGTCTG |
| IL-6 | mouse | Forward | GCCTTCTTGGGACTGATGCT |
|  |  | Reverse | GGTCTGTTGGGAGTGGTATCC |
| IL-1β | mouse | Forward | CACTACAGGCTCCGAGATGAACAAC |
|  |  | Reverse | TGTCGTTGCTTGGTTCTCCTTGTAC |
| MCP | mouse | Forward | TAAAAACCTGGATCGGAACCAAA |
|  |  | Reverse | GCATTAGCTTCAGATTTACGGGT |
| KIM-1 | mouse | Forward | CTGCTGCTACTGCTCCTTGTGAG |
|  |  | Reverse | CCACGCTTAGAGATGCTGACTTCC |
| TNFα | human | Forward | AGCCCTGGTATGAGCCCATCTATC |
|  |  | Reverse | TCCCAAAGTAGACCTGCCCAGAC |
| IL-6 | human | Forward | AGACAGCCACTCACCTCTTCAG |
|  |  | Reverse | TTCTGCCAGTGCCTCTTTGCTG |
| IL-1β | human | Forward | GCCAGTGAAATGATGGCTTATT |
|  |  | Reverse | AGGAGCACTTCATCTGTTTAGG |
| MCP | human | Forward | TAAAAACCTGGATCGGAACCAAA |
|  |  | Reverse | GCATTAGCTTCAGATTTACGGGT |
| Mt COX1 | human | Forward | ATTCATCGGCGTAAATCTAA |
|  |  | Reverse | AGGCTTCTCAAATCATGAAA |
| Mt ND1 | human | Forward | CCTCGTAGTAACAGCCATTC |
|  |  | Reverse | TTGAAGTCCTTGAGAGAGGA |
| 18s | human | Forward | GAGTCAACGGATTTGGTCGT |
|  |  | Reverse | TTGATTTTGGAGGGATCTCG |


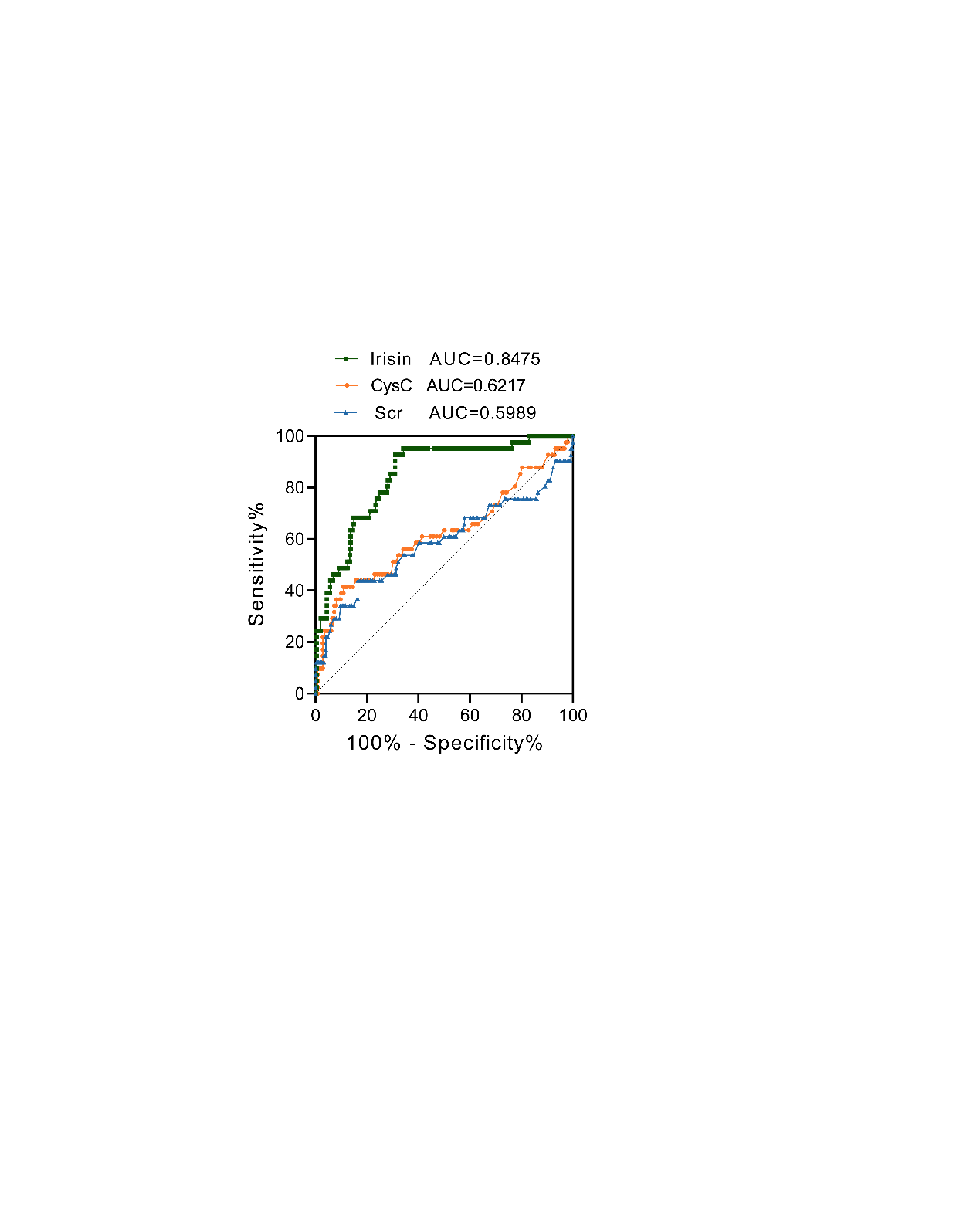


**Figure S1. Receiver operating curve analysis for irisin (green), CysC (orange) and Scr (blue) in the serum of patients undergoing CAG or PCI (n=290).**


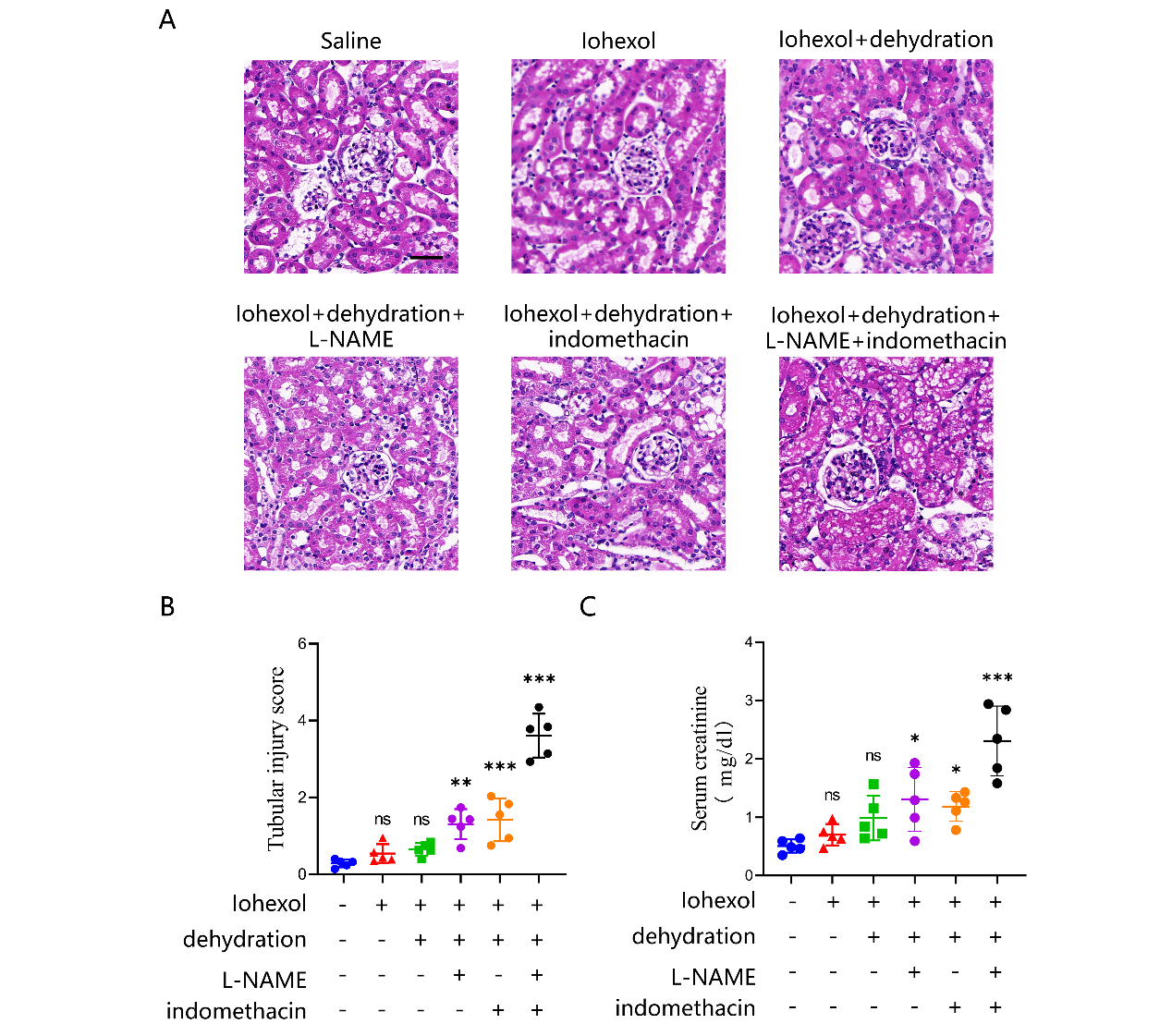


**Figure S2. Experimental induction of CI-AKI in mice (n=5).** A. Representative images of HE-stained kidney sections from Saline, Iohexol (5gI/kg), Iohexol + dehydration, Iohexol + dehydration + L-NAME (10mg/kg), Iohexol + dehydration + indomethacin (10mg/kg), and Iohexol + dehydration + indomethacin + L-NAME mice. Scale bars, 100 μm. B. The quantification of tubular injury based on the HE staining. C. The serum creatinine levels of the six groups. **P* < 0.05, ***P* < 0.01, and ****P* < 0.001. ns, no significance.


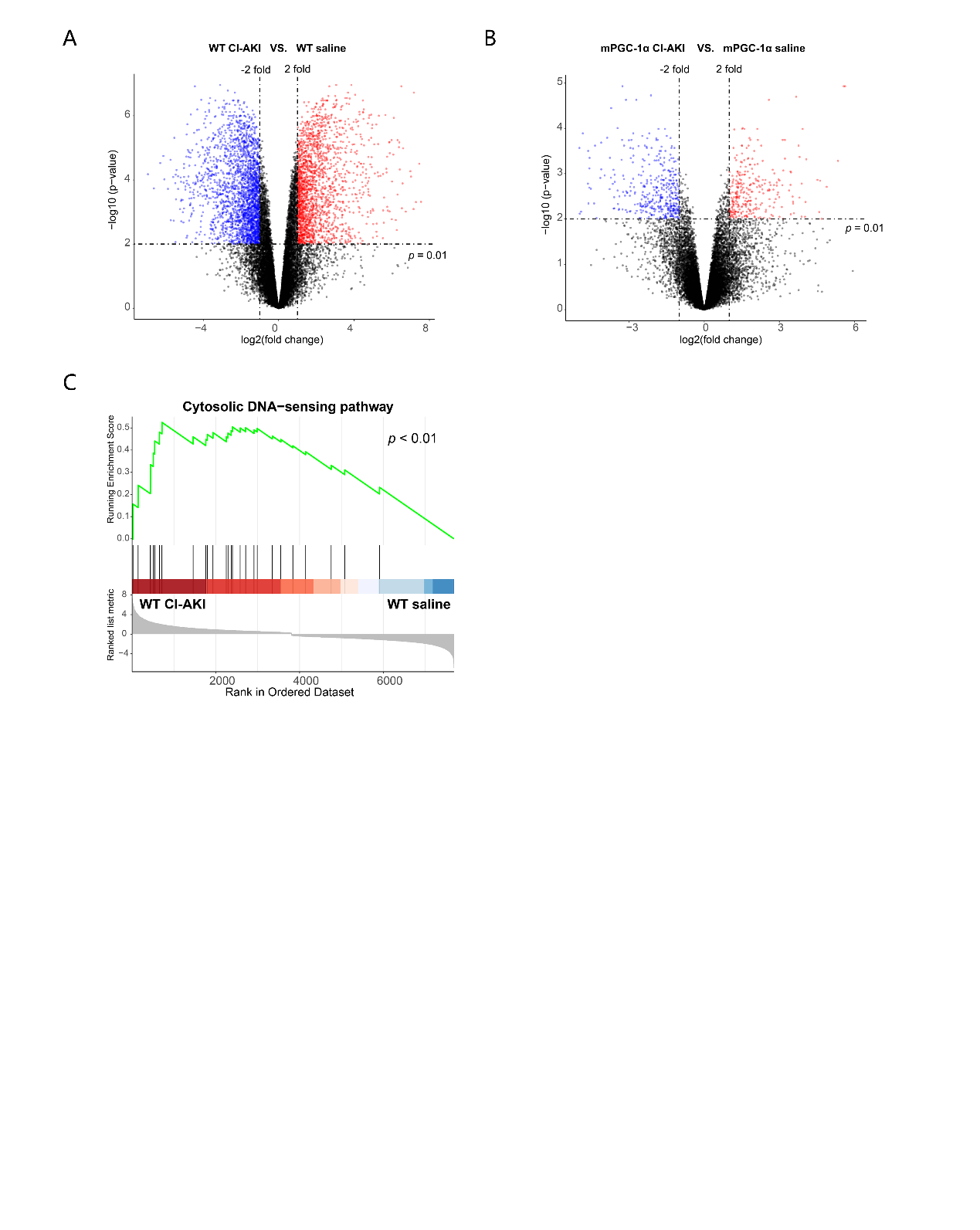


**Figure S3. The RNA-seq analyses of kidney tissues of WT saline, WT CI-AKI, mPGC-1α saline, and mPGC-1α CI-AKI mice (n=4).** A. volcano plot showing the differentially expressed genes between WT saline and WT CI-AKI groups. B. volcano plot showing the differentially expressed genes between mPGC-1α saline and mPGC-1α CI-AKI groups. C. GSEA plot showing the enrichment score of gene sets in the RNA-seq data of kidneys.


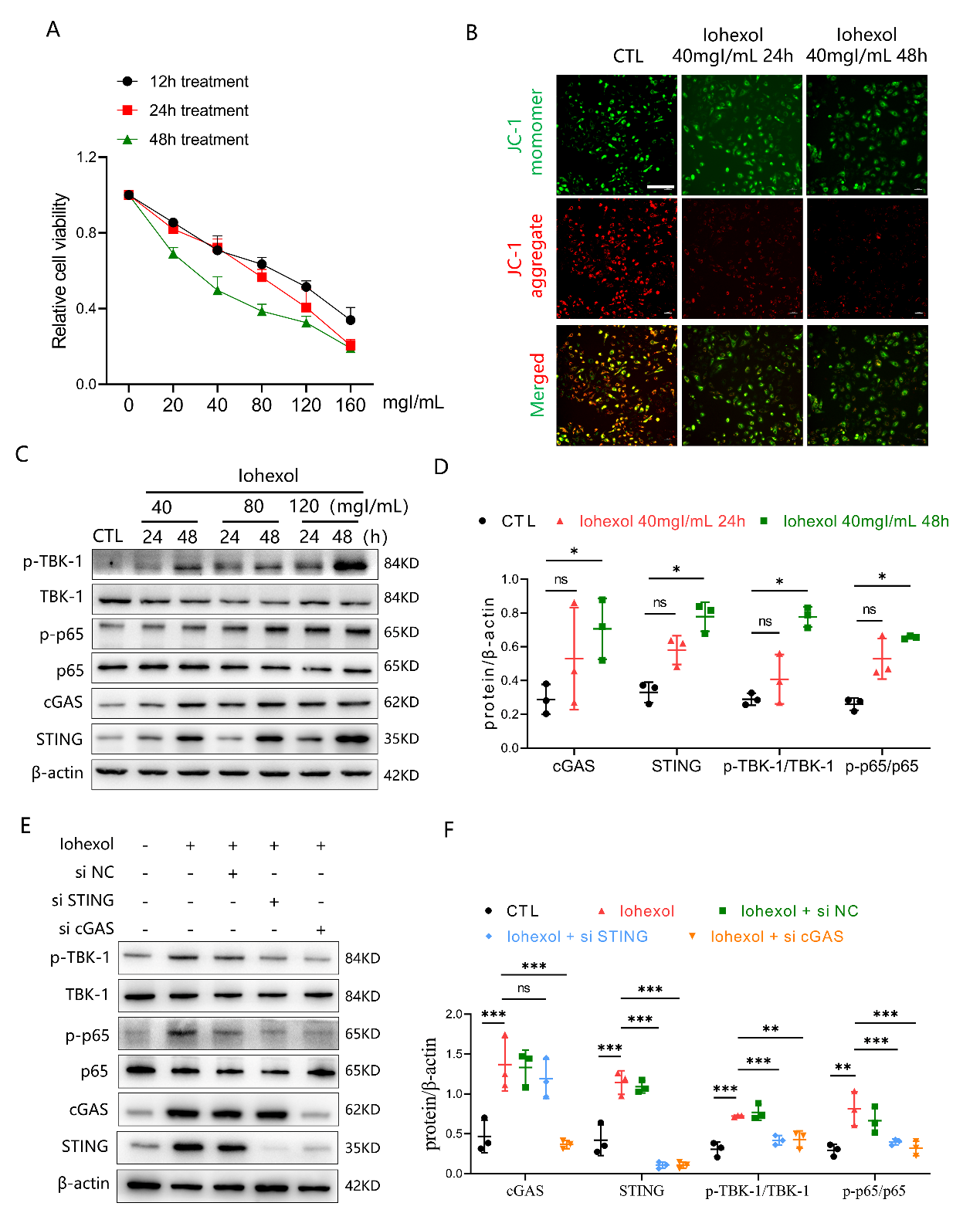


**Figure S4. cGAS-STING activation mediates cell injury and inflammatory responses induced by iohexol.** (A) TECs were treated with 0, 20, 40, 80, 120, 160 mgl/mL iohexol for 12 h, 24 h, 48 h respectively. Then the cell viability was examined by CCK-8 assays (n=3). (B) Immunofluorescence of the mitochondrial membrane potential in TECs labeled with JC-1. (C and D) Representative western blot images (C) and quantification analysis (D) of protein expression of cGAS, STING, p-TBK-1, TBK-1, p-p65, and p65 in TECs treated with iohexol (n=3). (E and F) Western blot analysis showing the effects of si-STING and si-cGAS on the expression of proteins related to cGAS-STING signaling pathway in TECs (n=3). Data are presented as means ± SD. One-way ANOVA with Tukey’s post hoc test was used for statistical analysis. **P* < 0.05, ***P* < 0.01, and ****P* < 0.001. ns, no significance.


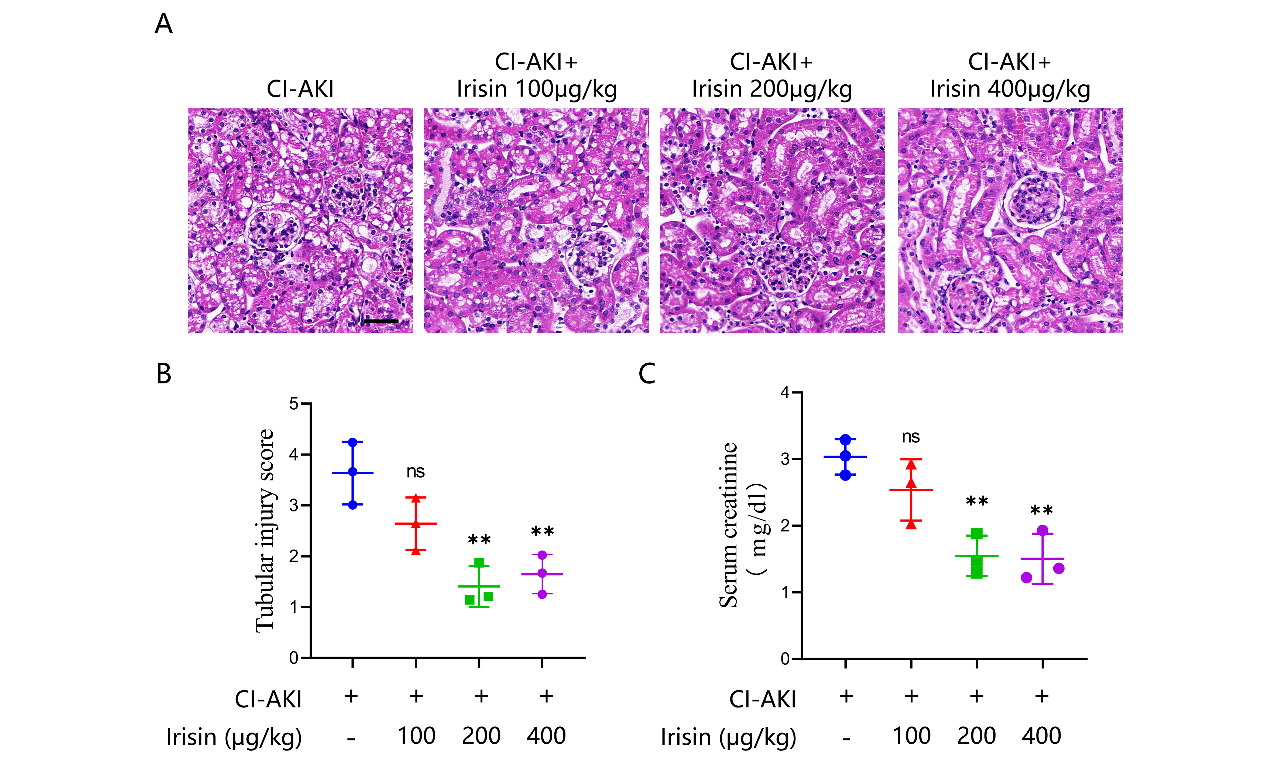


**Figure S5.** **Irisin treatment alleviates CI-AKI (n=3).** A. Mice were concurrently treated with irisin or vehicle 24 hours before iohexol exposure. Representative images of HE-stained kidney sections from CI-AKI, CI-AKI + Irisin (100 μg/kg), CI-AKI + Irisin (200 μg/kg), CI-AKI + Irisin (400 μg/kg) mice. Scale bars, 100 μm. B. The quantification of tubular injury based on the HE staining. C. Effects of irisin on serum creatinine. ***P* < 0.01. ns, no significance.


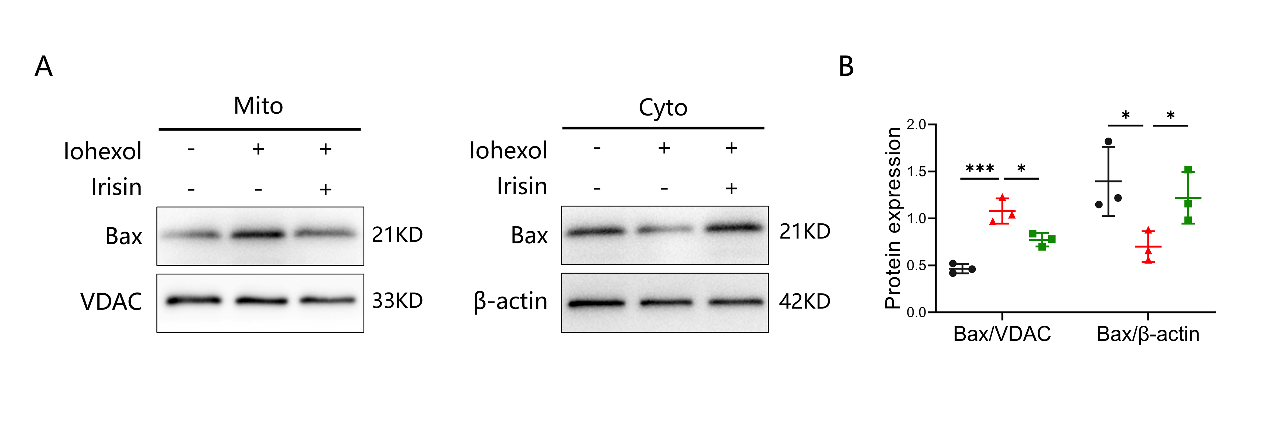


**Figure S6. Irisin decreases Bax mitochondrial translocation in TECs treated with iohexol.** (A and B) Representative western blot and quantitative data showing the effects of irisin on Bax protein abundance from mitochondria and cytoplasm in TECs with iohexol exposure (n=3). **P* < 0.05, and ****P* < 0.001.


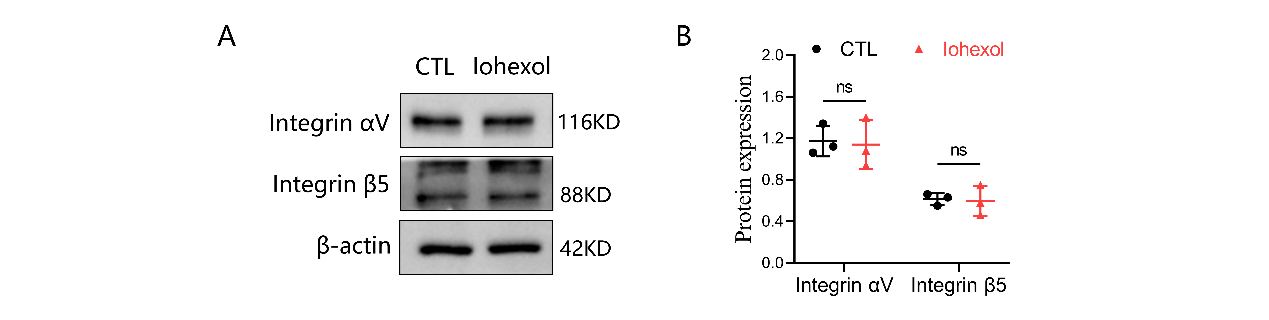


**Figure S7.** **The protein expressions of integrin αV and integrin β5 in TECs remain unaltered following iohexol stimulation.** (A and B) Representative western blot and quantitative data showing the effects of iohexol exposure on the protein expressions of integrin αV and integrin β5 (n=3). ns, no significance.
